# Incompatible erythrocyte transfusion with lipopolysaccharide induces acute lung injury in a novel rat model

**DOI:** 10.1371/journal.pone.0230482

**Published:** 2020-04-20

**Authors:** Magdielis Gregory Rivera, Alana C. Sampson, Pamela S. Hair, Haree K. Pallera, Kaitlyn G. Jackson, Adrianne I. Enos, Turaj Vazifedan, Alice L. Werner, Corinne L. Goldberg, Frank A. Lattanzio, Kenji M. Cunnion, Neel K. Krishna

**Affiliations:** 1 Department of Pediatrics, Eastern Virginia Medical School, Norfolk, Virginia, United States of America; 2 Department of Microbiology and Molecular Cell Biology, Eastern Virginia Medical School, Norfolk, Virginia, United States of America; 3 Children’s Hospital of The King’s Daughters, Norfolk, Virginia, United States of America; 4 Children’s Specialty Group, Norfolk, Virginia, United States of America; 5 American Red Cross, Durham, North Carolina, United States of America; 6 Department of Physiological Sciences, Eastern Virginia Medical School, Norfolk, Virginia, United States of America; Max Delbruck Centrum fur Molekulare Medizin Berlin Buch, GERMANY

## Abstract

Acute transfusion reactions can manifest in many forms including acute hemolytic transfusion reaction, allergic reaction and transfusion-related acute lung injury. We previously developed an acute hemolytic transfusion reaction rat model mediated by transfusion of incompatible human erythrocytes against which rats have preexisting antibodies resulting in classical complement pathway mediated intravascular hemolysis. In this study, the acute hemolytic transfusion reaction model was adapted to yield an acute lung injury phenotype. Adolescent male Wistar rats were primed in the presence or absence of lipopolysaccharide followed by transfusion of incompatible erythrocytes. Blood was collected at various time points during the course of the experiment to determine complement C5a levels and free DNA in isolated plasma. At 4 hours, blood and lung tissue were recovered and assayed for complete blood count and histological acute lung injury, respectively. Compared to sham animals or animals receiving increasing amounts of incompatible erythrocytes (equivalent to a 15–45% transfusion) in the absence of lipopolysaccharide, lungs of animals receiving lipopolysaccharide and a 30% erythrocyte transfusion showed dramatic alveolar wall thickening due to neutrophil infiltration. C5a levels were significantly elevated in these animals indicating that complement activation contributes to lung damage. Additionally, these animals demonstrated a significant increase of free DNA in the blood over time suggestive of neutrophil extracellular trap formation previously associated with transfusion-related acute lung injury in humans and mice. This novel ‘two-hit’ model utilizing incompatible erythrocyte transfusion in the presence of lipopolysaccharide yields a robust acute lung injury phenotype.

## Introduction

Acute transfusion reactions (ATR) are estimated to occur in nearly one-fifth of total transfusions with approximately 0.5% resulting in life-threatening reactions [1]. Acute hemolytic transfusion reactions (AHTR) represent a subset of these reactions and can manifest as a broad clinical presentation from mild and transitory signs and symptoms to serious cases of AHTR leading to shock, renal failure, disseminated intravascular coagulation and death [1–4]. As there are currently no specific therapeutic interventions to directly inhibit AHTRs, current standard of care is primarily supportive in nature and dictated by the severity of the clinical presentation. Preventive measures to reduce the incidence of AHTR have greatly reduced the number of transfusion related adverse events, however transfusion reactions still occur [5].

The complement system plays a key role in AHTRs such as acute intravascular hemolytic transfusion reaction (AIHTR) [1,2,4,6]. AIHTR occurs when transfused incompatible erythrocytes are bound by host antibodies in the serum of the recipient initiating classical complement pathway activation which leads to C3b opsonization and subsequent intravascular hemolysis of the transfused cells via the membrane attack complex (MAC). We have previously developed a rat model of AIHTR utilizing transfusion of mismatched erythrocytes mimicking ABO incompatibility. In this model, the rat species has preexisting antibodies to the A antigen [7] of human erythrocytes resulting in a robust AITHR phenotype after transfusion of human erythrocytes from a type A or type AB donor [8]. This AIHTR model causes antibody-initiated classical complement pathway activation including neutrophilia [8,9]. The pathogenic aspects of antibody-initiated complement activation and mobilization of neutrophils suggested that this model could be modified to yield an acute lung injury (ALI) phenotype, if the inflammatory response were directed towards the lungs. Here we report adaption of this transfusion model to induce a neutrophil-mediated ALI by infusion of lipopolysaccharide (LPS) into rats followed by 30% human erythrocyte transfusion. Development of this novel ‘two-hit’ model provides a suitable platform to probe pathogenesis in a transfusion-induced robust neutrophil-mediated ALI phenotype and potentially test the efficacy of immunomodulators in this setting.

## Materials and methods

### Ethics statement and animal welfare

Animal Research: Animal research was approved by the EVMS IACUC, protocol #18–001. For euthanasia of rats, animals deeply anesthetized with a cocktail of ketamine/acepromazine were subsequently subject to isofluorane inhalation followed by decapitation by guillotine. Adolescent male Wistar rats (200–250 g) were purchased from Hilltop Lab Animals (Scottdale, PA, USA) with indwelling jugular catheters. Care and handling of the animals were in accord with NIH guidelines.

Human subjects research: Human subjects research was approved by the Eastern Virginia Medical School (EVMS) IRB, protocol #02-06-EX 0216. Written consent was obtained. A healthy human volunteer (type AB+) donating whole blood was used as the source of purified human erythrocytes.

### Human erythrocyte purification

Human erythrocytes from an AB+ donor were acquired the day before the animal experiments and processed as described previously [8]. Briefly, 20 mL of human blood was purified on a Histopaque (Sigma-Aldrich, Saint Louis, MO, USA) gradient by centrifugation. The erythrocytes were then separated from white blood cells and platelets and resuspended in saline. Rats (200g) have a nominal circulating blood volume of 14 mL with a nominal 40% hematocrit. For transfusion, 2 mL of human erythrocytes at 80% hematocrit was administered, which results in a 30% transfusion to the rats.

Histopaque gradient purification of erythrocytes is not commonly utilized in clinical practice as leukoreduction filters are the gold standard for removing contaminating white blood cells. To exclude the possibility that human granulocytes were present in the erythrocyte preparations, the purified erythrocyte preparations were analyzed for contaminating granulocytes on a hemocytometer. Visual inspection of multiple fields of erythrocyte preparations with final cell counts of 8 x 10^10^ cells/ml did not reveal any viable contaminating human granulocytes. In parallel to analysis on the hemocytometer purified erythrocytes were also analyzed by cytospin. One granulocyte was observed for every 2–3 high powered fields. Each view had on average 100 cells. Visual inspection of the rare granulocyte showed the cells not to be intact suggesting they were non-viable.

### Animal experiments

**Fig 1:**
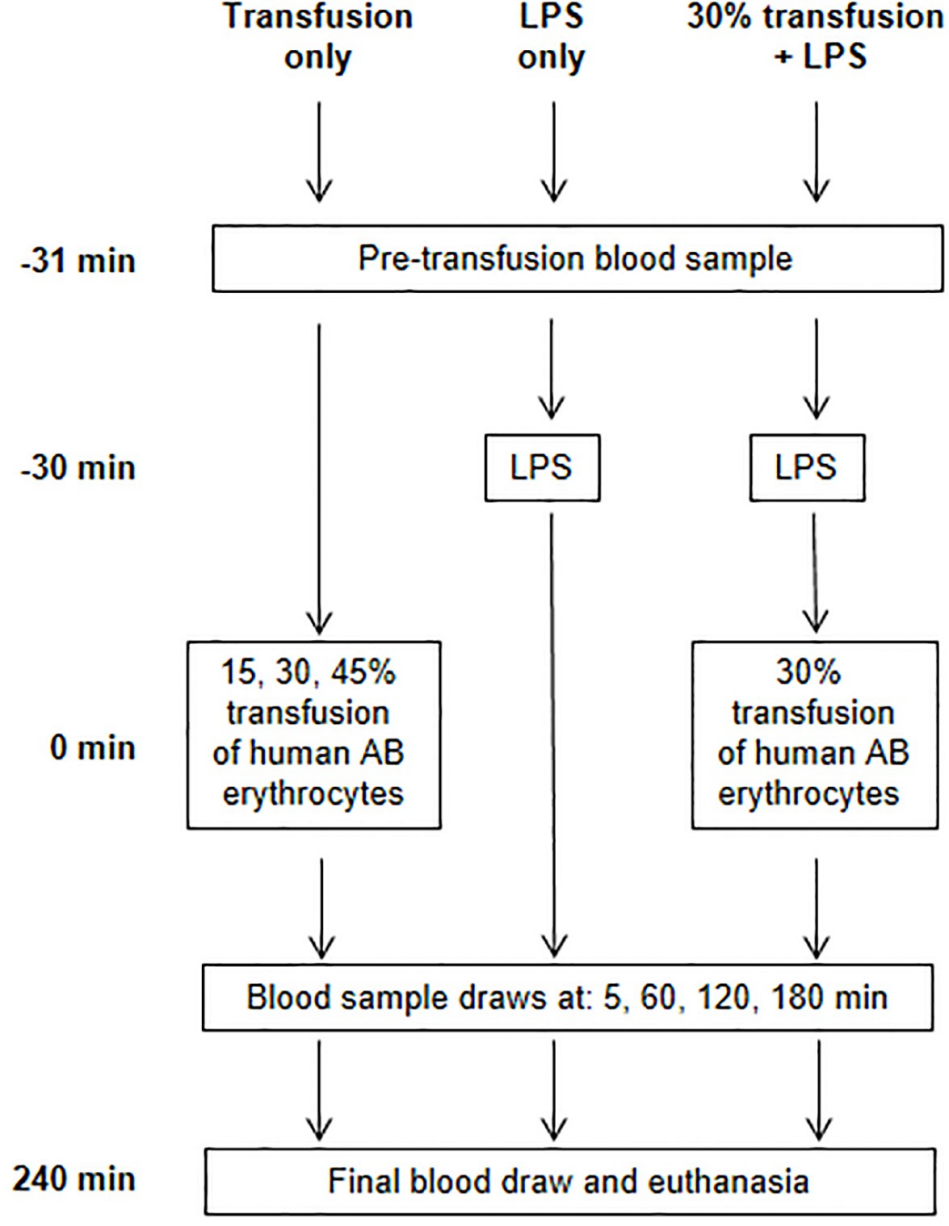
Experimental design and study arms.

**Fig 2:**
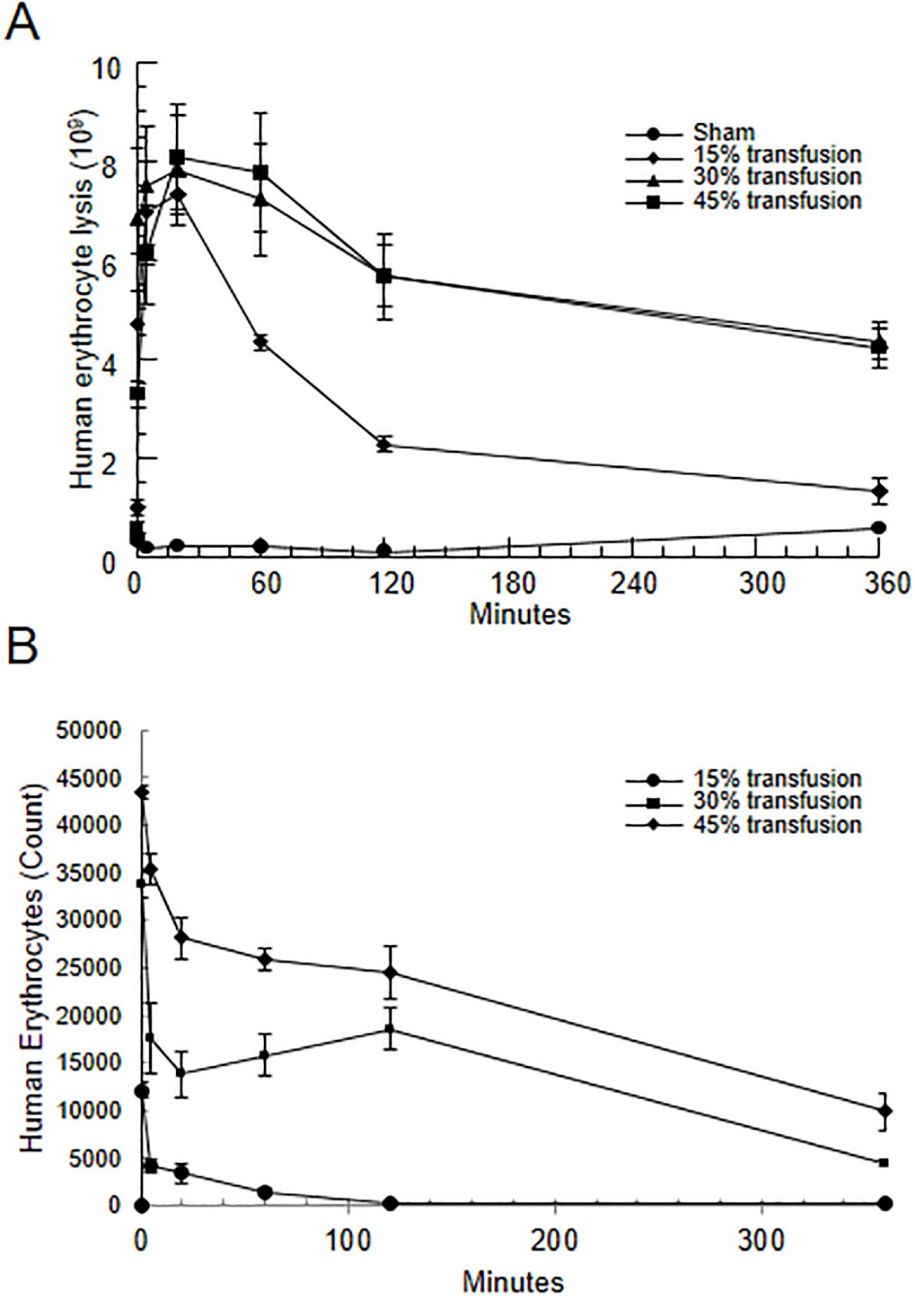
Optimization of human erythrocyte transfusion into rats. (A) Free hemoglobin present in rat plasma (expressed as an equivalent number of lysed erythrocytes) collected before transfusion (0) or 0.5, 5, 20, 60, 120 or 360 min after 15 (n = 3), 30 (n = 3) or 45% (n = 3) transfusion of human erythrocytes was measured by spectrophotometry. One group of sham animals (n = 3) was analyzed as well. (B) Absolute cell count of surviving human erythrocytes from 15 (n = 3), 30 (n = 3) or 45% (n = 3) transfusion of human erythrocytes were detected using FITC-conjugated anti-human CD235a (glycophorin A) monoclonal antibody at 0.5, 5, 20, 60, 120 and 360 min after transfusion as measured by flow cytometry. Clearance kinetics were standardized to injected erythrocytes at baseline (0 min). Data are means and standard error of the mean.

To establish the ALI model, we modified our previously published AHTR rat model (Fig 1) [8,9]. Varying amounts of human erythrocytes (15, 30 or 45%) were initially transfused into rats to optimize the model. For all procedures, rats were sedated with ketamine (McKesson, Las Colinas, TX, USA) and acepromazine (Patterson Veterinary, Saint Paul, MN, USA) at (75/2.5 mg/kg IP) throughout the course of the experiment with monitoring of vital signs. Animals were allowed to wake up between blood draws and resedated before the terminal blood draw. Groups of rats received transfusion of human erythrocytes intravascularly through the indwelling jugular catheter. Blood samples were collected into K_2_EDTA microtainer tubes (Becton Dickinson, Franklin Lakes, NJ, USA) from the animals prior to transfusion and then at 0.5, 5, 20, 60, 120 and 360 min after transfusion. These samples were centrifuged at 2,655 × *g* for 5 min to separate out the plasma and sediment the cells. Plasma was aliquoted and the cell pellet was processed separately as described below. Based on pilot experiments with varying amounts of human erythrocytes (15–45%), transfusion of 30% human erythrocytes produced robust complement-mediated hemolysis over 6 hours and was chosen for the ALI model (Fig 2A and 2B).

To generate a vigorous ALI phenotype, rats were sedated as above and lipopolysaccharide (LPS, from *Salmonella enterica* serotype enteritidis, 2 mg/kg [MilliporeSigma, Burlington, MA, USA]) was administered intravascularly through the indwelling jugular catheter as the ‘first-hit’ similar to previously reported ALI models [10,11]. This was followed 30 minutes later by 30% ABO mismatched erythrocyte transfusion as the ‘second-hit’ (Fig 1). Sham animals and animals receiving either LPS or the erythrocyte transfusion alone served as controls. Blood samples were collected prior to LPS or erythrocyte administration (time 0) and at 5, 60, 120, 180 and 240 minutes after erythrocyte transfusion (Fig 1). The 0, 5 and 240 minute sample were used for analysis of blood chemistries (SuperChem and CBC, Antech Diagnostics, Lake Success, NY, USA). Additionally, all blood samples were analyzed for C5a levels and free DNA concentration as described below. Upon completion of the final blood draw, the animals were euthanized using isofluorane (McKesson) and guillotine. A necropsy was completed to collect organs for histopathology. In a subset of animals, lungs were weighed and then stored in formalin or frozen at -70°C. Lungs from a separate subset of animals were weighed and dried in an oven at 65°C for 3 days and then reweighed.

Compared to animals receiving 15–45% incompatible erythrocyte transfusion or LPS alone, rats receiving LPS+30% erythrocyte transfusions appeared lethargic and displayed reduced activity. However, the treatment group animals were not observed to be in pain or suffering and there was no increase in mortality in this group of animals compared with sham animals during the course of the experiment.

### Hemoglobin measurements

Plasma generated from the above experiments was analyzed for free hemoglobin using spectrophotometry, as described previously [8,9]. Donor erythrocytes were hemolyzed with water to generate a standard curve from which the amount of hemolyzed erythrocytes in each sample was calculated with respect to the free hemoglobin measurements.

### Flow cytometry

Flow cytometry was performed using a FACSCalibur flow cytometer (Becton Dickinson, Franklin Lakes, NJ, USA) with DXP 8 Color 488/637/407 upgrade (Cytek Development, Freemont, CA, USA). The data was acquired using Cytek FlowJo CE version 7.5.110.6. Approximately 1x10^5^ events, selected for erythrocytes, per sample were gathered for single labeled flow, respectively. Data was analyzed using FlowJo X version 10.0.7r2 (FlowJo, LLC, Ashland, OR, USA).

For single labeled flow, the cells collected after separating the plasma were washed, diluted and stained with FITC-conjugated anti-human CD235a (glycophorin A, [eBioscience-ThermoFisher Scientific, Waltham MA, USA]) at 1:200 in GVBS—^-^ (veronal-buffered saline (VBS) with 0.1% gelatin, 0.01 mol/L EDTA (ethylenediaminetetraacetic acid)) for 20 min while shaking at room temperature to minimize agglutination [8,9]. An antibody control consisted of mouse IgG2b Iso-control FITC at 1:200 (eBioscience- ThermoFisher Scientific).

### Lung injury score

Lung tissue stained with hematoxylin and eosin (H&E) was analyzed by a physician blinded to the experimental groups. Ten random microscopy fields were scored for each rat; five fields for each lung (left and right). Neutrophil infiltration and cell wall thickening were scored on a scale of 0–4: 0 = normal lungs, 1 = minor lung involvement, 2 = moderate lung involvement, 3 = serious lung involvement, 4 = severe lung involvement.

### Plasma C5a measurements

C5a levels were measured from plasma samples by ELISA, according to the manufacturer’s instructions (LSBio, Seattle, WA). Briefly, diluted rat plasma samples were added to wells pre-coated with C5a antibodies. The plate was incubated for 90 minutes at 37°C, then any unbound components were removed by washing. One hundred μL of a biotin-conjugated C5a detection antibody (diluted 1:100) was added and incubated for 60 minutes at 37°C. Next, 100 μL of streptavidin-horseradish peroxidase (HRP) conjugate (diluted 1:100) was added and incubated for 30 minutes at 37°C. Following washing, 90 μL of 3,3’,5,5’-tetramethylbenzidine (TMB) substrate was added for 30 minutes at 37°C. The reaction was stopped with the addition of 50 μL of sulfuric acid and absorbance measured at 450 nm using a BioTek microplate reader. Sample concentrations were calculated using a C5a standard curve made with 1:2 serial dilutions.

### Plasma DNA measurements

Free DNA was measured by PicoGreen in rat plasma samples as previously described [12]. Briefly, plasma samples were diluted in 10 mM Tris-HCl, 1 mM EDTA, pH 8.0 (TE) buffer and 50uL of each sample was added to the wells along with 50uL of a 1:200 dilution of PicoGreen (Life Technologies, Carlsbad, CA, USA) and incubated at room temperature for 10 minutes, protected from light. A DNA standard curve was prepared in TE Buffer. The fluorescence was then read at an excitation wavelength of 485nm and an emission wavelength of 520nm using a BioTek microplate reader. All free DNA measurements were done in triplicate.

### Statistical analysis

Means and standard errors were calculated from independent experiments and statistical comparisons were made using one way ANOVA, followed by Student t-test. A Kruskal-Wallis test, followed by Mann-Whitney test, was conducted to compare the level of lung injury and wet lung weight between groups. The generalized linear model was used to compare c5a and DNA between groups. All statistical tests were performed using SPSS 26 (Chicago, IL). All tests were two-sided with the significant level set at 0.05. The Results of the ANOVA analysis are provided as supplementary data (S1 File).

## Results

### Optimization of incompatible erythrocyte transfusion

Previous work in our laboratory established a rat model of AIHTR in which transfusion of 15% mismatched erythrocytes resulted in intravascular hemolysis and acute kidney injury [8]. In this model, naturally circulating anti-A antibodies in Wistar rats [7] initiate classical complement activation and hemolysis of the transfused erythrocytes. To ascertain if the AIHTR model could be adapted to mimic an ALI phenotype, ascending doses of mismatched erythrocyte transfusions (15, 30 or 45%) were initially tested and it was determined that a 30% transfusion produces near maximal amounts of complement-mediated hemolysis as measured by free hemoglobin in circulation (Fig 2A). Similar amounts of hemolysis, as measured by free hemoglobin in plasma, seen at 30% and 45% transfusion suggested that the capacity for antibody-initiated complement-mediated hemolysis is exceeded above 30% transfusion. As expected, the number of transfused erythrocytes in circulation increased with the amount of cells transfused (15, 30 and 45%) as assessed by flow cytometry (Fig 2B). Free hemoglobin demonstrates a longer residence in circulation compared with mismatched erythrocytes as its elimination requires scavenging by haptoglobin and excretion via the kidney over a period of hours. The free hemoglobin curves suggest that in the case of the 30 and 45% transfusion, the large amount of free hemoglobin saturates circulating haptoglobin and other hemoglobin scavengers leading to a slower clearance over time compared to the 15% transfusion. In contrast, the incompatible erythrocytes are both destroyed by complement-mediated lysis and sequestered in the spleen and liver as we have previously reported for this model [8]. Given the intermediated phenotype of hemolysis, robust without saturating hemoglobin elimination, with the 30% erythrocyte transfusion compared to the 15 and 45% transfusions, the 30% erythrocyte preparation was chosen for the ALI model.

### LPS induces a decrease in circulating leukocytes

**Fig 3:**
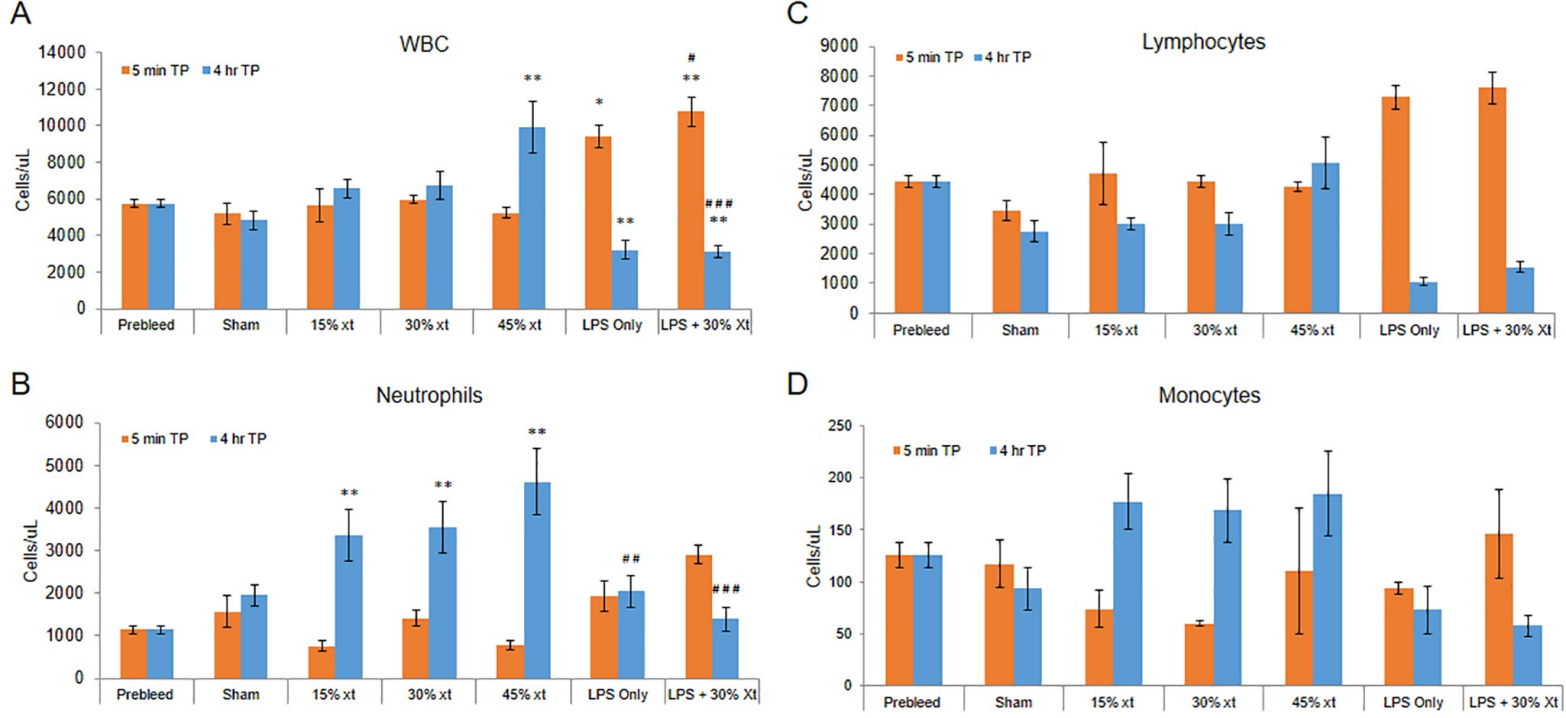
LPS induces leukopenia. Blood was collected from rats before addition of LPS or transfusion (0 min, pre-bleed time point (tp)), (n = 64). Five minutes and 4 hour later, blood was again collected from sham rats (5 min (n = 5) and 4 hours (n = 8)), rats transfused with 15% (5 min (n = 3) and 4 hours (n = 6)), 30% (5 min (n = 3) and 4 hours (n = 9)) or 45% (5 min (n = 3) and 4 hours (n = 6)) human erythrocyte transfusion (xt), LPS only (5 min (n = 4) and 4 hours (n = 10)) or LPS+30% (5 min (n = 3) and 4 hours (n = 13)) transfusion. (A) White blood cells (WBCs). (B) Neutrophils. (C) Monocytes. (D) Lymphocytes. Data are means and standard error of the mean. Statistical analysis was performed using an ANOVA followed by Student *t*-test. * denotes *P ≤* 0.02, ** denotes *P ≤* 0.005, respectively, compared to the pre-transfusion control at the corresponding timepoint. # denotes *P < 0*.*05*, ## denotes *P < 0*.*01 and* ### denotes *P < 0*.*001*, respectively, compared to 30% xt at the corresponding timepoint.

To ascertain the effect of leukocyte mobilization after erythrocyte transfusion, blood levels of these cells were determined prior to transfusion of 15, 30 or 45% incompatible erythrocytes as well as 5 minutes and four hours after erythrocyte transfusion. At 5 minutes post-transfusion, white blood cells (WBCs) were at levels similar to time 0 and sham-treated animals (P = 0.96). At four hours post-transfusion, WBC levels were slightly increased for animals receiving 15 and 30% erythrocyte transfusion whereas animals that received the 45% transfusion were significantly elevated compared to time 0 (P<0.001) (Fig 3A). Published animal models of ALI typically utilize a ‘two-hit’ model that directs the inflammatory response to the lungs (reviewed in references [13,14]). A common method of inducing the ‘first-hit’ is infusion of LPS which will initially encounter the capillary beds of the lungs likely priming them for overt damage by the ‘second hit’ [10,11]. A 2 mg/kg LPS intravenous infusion given alone (P = 0.005) or followed by 30% erythrocyte transfusion (P<0.001) resulted in a marked increase in blood levels of WBCs at 5 minutes compared to time 0 controls (Fig 3A). In contrast, a significant reduction in circulating levels of WBC was observed at 4 hours compared to pre-transfusion controls for both LPS only (P<0.001) and LPS+30% erythrocyte transfusion (P<0.001) indicative of LPS-induced removal from circulation after initial mobilization into the bloodstream (Fig 3A). The increase in WBCs at 5 min and reduction at 4 hours was also significant when comparing the 30% erythrocyte transfusion group to animal receiving LPS+30% erythrocyte transfusion (P = 0.017 and P<0.001, respectively).

Analysis of neutrophils in sham animals or animals receiving 15–45% erythrocyte transfusion revealed that levels of these cells did not significantly change compared to the pre-bleed controls when analyzed 5 minutes after transfusion (P = 0.53) (Fig 3B). In contrast, at 4 hours post-transfusion, animals receiving 15–45% erythrocyte transfusion had significantly increased number of neutrophils in circulation compared to time 0 controls (P<0.001). When the groups receiving LPS alone or LPS+30% mismatched erythrocytes were compared to animals receiving the 30% transfusion alone, a significant reduction of neutrophils (P = 0.009 and P < 0.001, respectively), was observed at 4 hours (Fig 3B). These results suggest that LPS is causing removal of WBCs from circulation. Analysis of lymphocytes revealed similar trends in levels of these cells between the groups as observed for WBC (compare Fig 3A and 3C) whereas monocyte levels tracked with neutrophils (compare Fig 3B and 3D). Interestingly, macrophages have previously been implicated in a mouse models of TRALI [11]. Taken together, this model demonstrates that LPS together with a mismatched transfusion induces an initial increase in WBC and neutrophil count at 5 minutes follow by a decrease in both after 4 hours suggesting initial mobilization into the bloodstream and then removal from circulation.

### LPS and 30% erythrocyte transfusion causes neutrophil-mediated ALI

**Fig 4:**
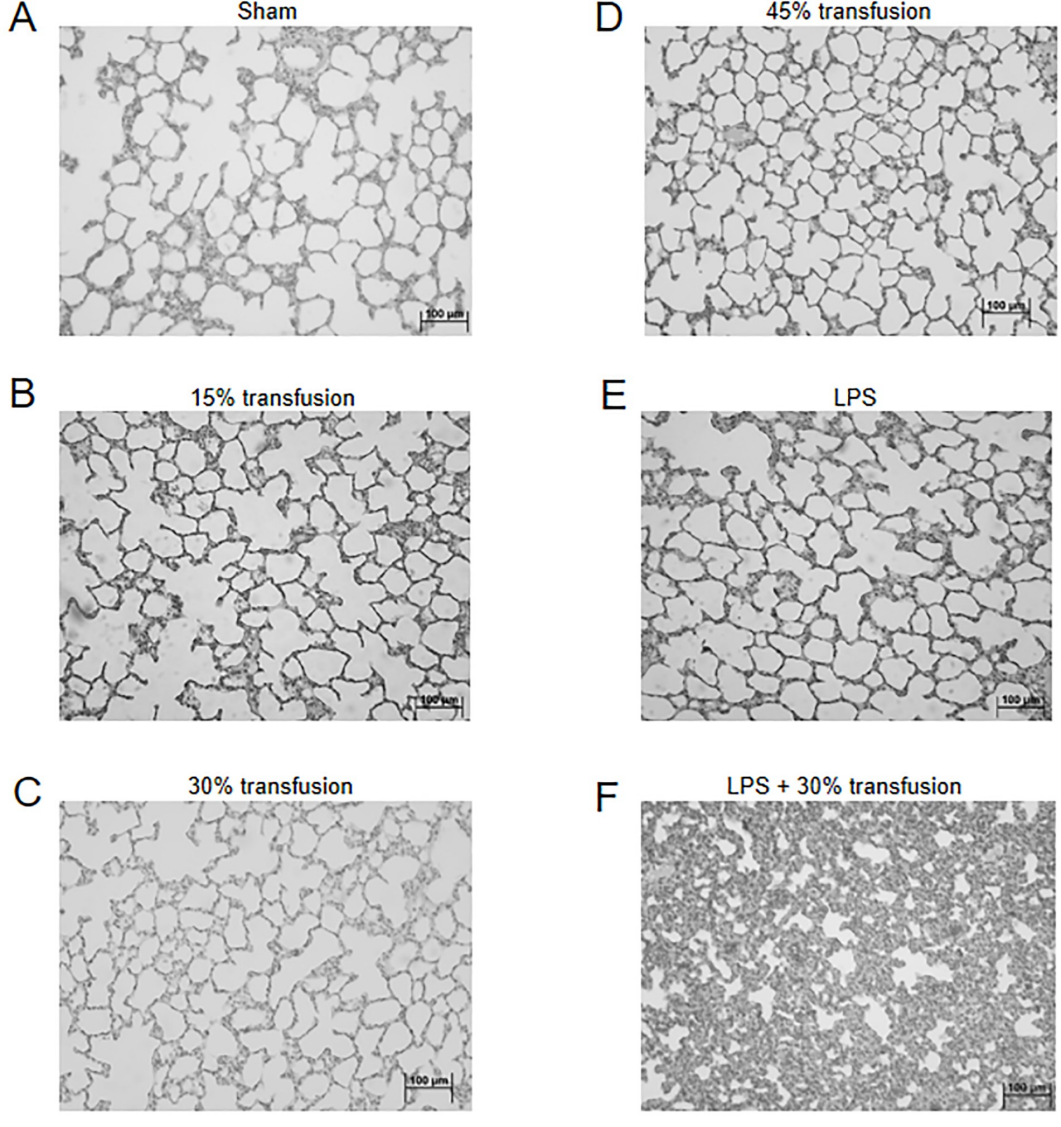
LPS+30% transfusion results in acute lung injury. Representative histology (H&E stain) of rat lungs. (A) Sham control. (B) 15% transfusion of human erythrocytes. (C) 30% transfusion. (D) 45% transfusion. (E) LPS only. (F) LPS+30% transfusion. Animals receiving transfusion in the absence of LPS and LPS alone demonstrated normal lung architecture as seen in sham treated animals whereas animals receiving LPS+30% transfusion showed severe neutrophil infiltration and thickening of the alveolar walls. Bar represents 100 μm. Tissues were observed with a microscope (BX50, Olympus) at a magnification of 20X at room temperature. Images were acquired with a digital camera (DP70, Olympus).

To evaluate the effect of erythrocyte transfusion in the absence and presence of LPS on lung tissue, lungs were isolated from a subset of the animals at four hours and tissues evaluated by H&E staining. Staining revealed that compared to sham animals, rats receiving 15, 30 and 45% erythrocyte transfusion did not demonstrate overt histologically identifiable lung damage (Fig 4A–4D). Similarly, LPS alone did not show any lung damage (Fig 4E). In contrast, rats receiving LPS followed by 30% erythrocyte infusion demonstrated dramatic lung damage at 4 hours post transfusion with areas of massive neutrophil infiltration into the alveolar walls (Fig 4F). Minimal lung edema was seen on histology for all groups (confirmed by our Pathologist).

**Fig 5:**
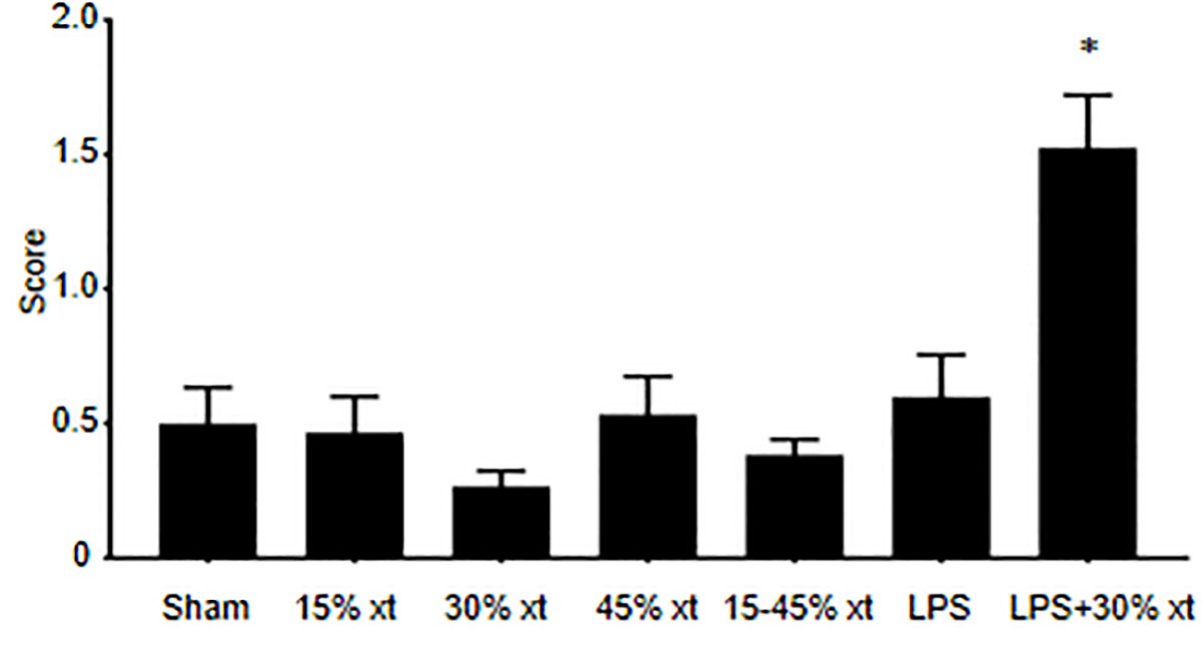
LPS+30% transfusion increases neutrophil-mediated lung injury. Blinded grading of H&E sections for neutrophil infiltration and cell wall thickening from sham (n = 20) animals and animals receiving 15 (n = 15), 30 (n = 30), 45% (n = 15) human erythrocyte transfusion (xt), LPS alone (n = 15) and LPS+30% transfusion (n = 20). Tissues were scored on a scale of 0–4: 0 = normal lungs, 1 = minor lung involvement, 2 = moderate lung involvement, 3 = serious lung involvement, 4 = severe lung involvement. Data are means and standard error of the mean. Statistical analysis was performed using a Kruskal-Wallis test, followed by Mann-Whitney test. * denotes *P* = 0.05 compared to all other experimental groups.

To ascertain the level of neutrophil-mediated lung injury in this model, a blinded grading of H&E sections for neutrophil infiltration and cell wall thickening from the different treatment groups was performed. Neutrophil infiltration and alveolar wall thickening were scored on a scale of 0–4 with a score of 0 indicating normal lungs and a score of 4 denoting severe lung injury. Animals receiving 15, 30, or 45% erythrocyte transfusion or LPS only showed low levels of lung damage similar to sham animals (Fig 5). In contrast, animals receiving LPS+30% transfusion demonstrated a significant increase in lung damage compared to the other groups including animals treated with LPS alone (P < 0.05). For the LPS+30% transfusion group the areas of severe disease with heavy neutrophil infiltration of the alveolar walls was interspersed with areas of relatively normal histology resulting in a mean score over ten random fields for each rat of 1.5. These results demonstrate that while LPS is required for neutrophil recruitment out of the bloodstream and initiation of lung damage, the LPS+30% transfusion results in a severe neutrophil mediated ALI phenotype compared to animals receiving LPS alone.

**Fig 6:**
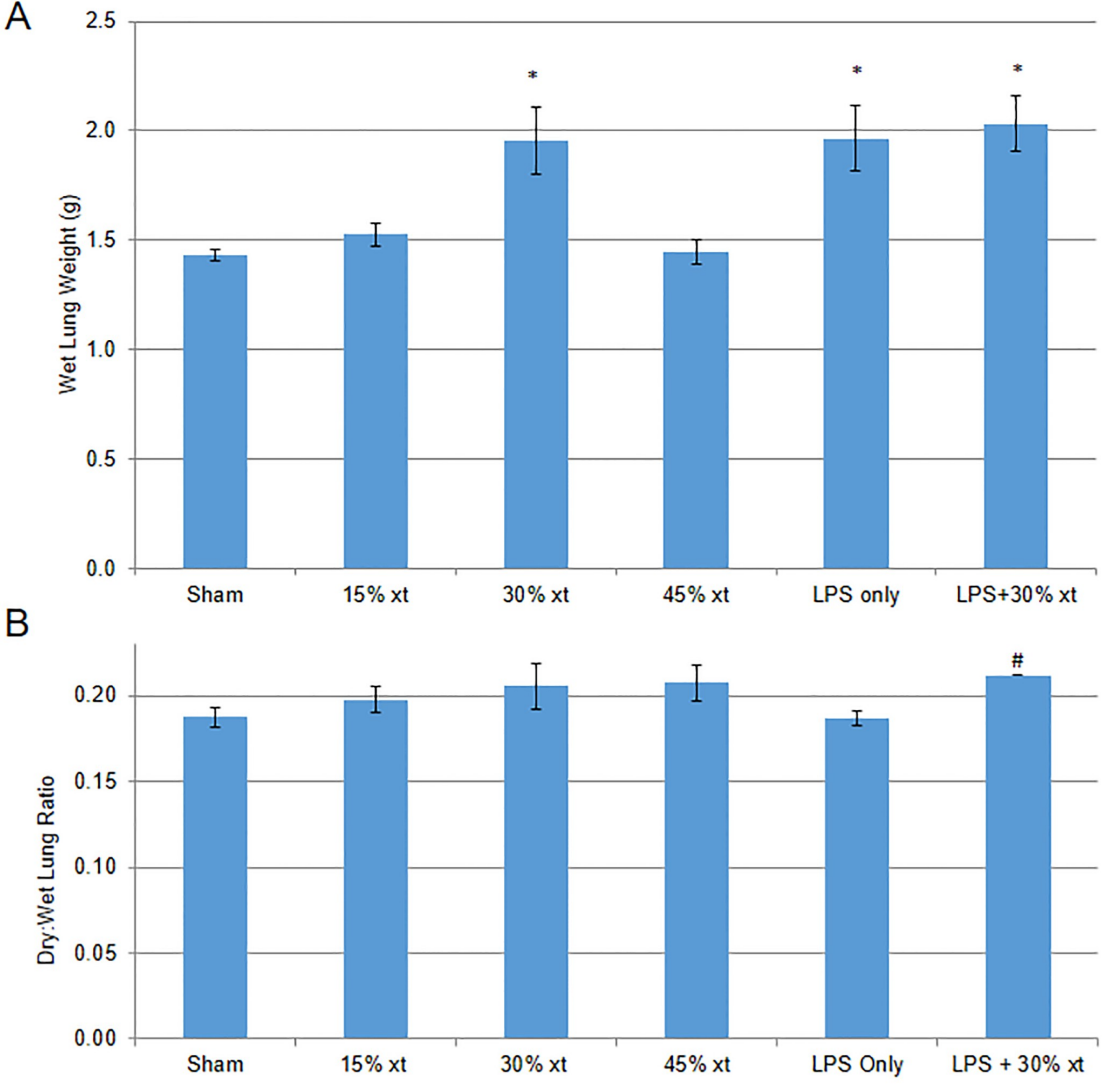
Thirty percent erythrocyte transfusion with LPS treatment increases lung weight. (A) Gross lung weights measured for sham animals (n = 6), rats receiving 15 (n = 3), 30 (n = 6) or 45% (n = 3) human erythrocyte transfusion (xt), LPS only (n = 7) or LPS+30% (n = 11) transfusion. (B) Wet and dry weights measured for sham animals (n = 8), rats transfused with 15 (n = 2), 30 (n = 3) or 45% (n = 3) human erythrocyte transfusion (xt), LPS only (n = 3) or LPS+30% (n = 3) transfusion and expressed as dry to wet ratio. Data are means and standard error of the mean. Statistical analysis was performed using an ANOVA followed by Student *t*-test. * denotes *P* < 0.01 compared to the sham. # denotes *P* < 0.05 compared to LPS only.

To evaluate the effect of erythrocyte transfusion in the absence and presence of LPS on lung tissue, lungs were isolated from a subset of the animals four hours after erythrocyte transfusion and weighed. Wet lung weight showed that animals receiving 30% erythrocyte transfusion, LPS alone or LPS+30% transfusion had a significant increase in lung weight of 34%, 36% and 41% compared with sham (P = 0.008, P = 0.009, P = 0.001, respectively) (Fig 6A). For a subset of animals, lungs were isolated four hours after erythrocyte transfusion, weighed, dried and then reweighed to calculate a dry to wet ratio. The heavy neutrophil infiltration in the alveolar walls together with the lack of intra-alveolar edema suggested that most of the increase in lung weight was due to increased cellular mass rather than edema. Thus, we calculated a dry:wet ratio to better isolate the contribution of increased cellular mass to increased lung weight (i.e., if most of the increased lung weight is due to cellular mass, then the dry:wet ratio will increase). Compared to sham animals, animals receiving 15%, 30% and 45% erythrocyte transfusion showed a trend to increase in dry:wet ratio over sham, however these changes were not statistically significant (Fig 6B). LPS treated animals showed no increase in dry:wet lung weight compared to sham, whereas animals receiving LPS+30% erythrocyte transfusion showed a significant increase in dry:wet ratio over animals treated with LPS only (P = 0.035). The increase in dry:wet ratio for rats receiving LPS+30% erythrocyte transfusion suggests that the increase in lung weight is due to increased cellular mass consistent with neutrophil infiltration of alveolar walls shown in the histological data (Fig 4F).

### LPS and 30% erythrocyte transfusion results in significant C5a production

**Fig 7:**
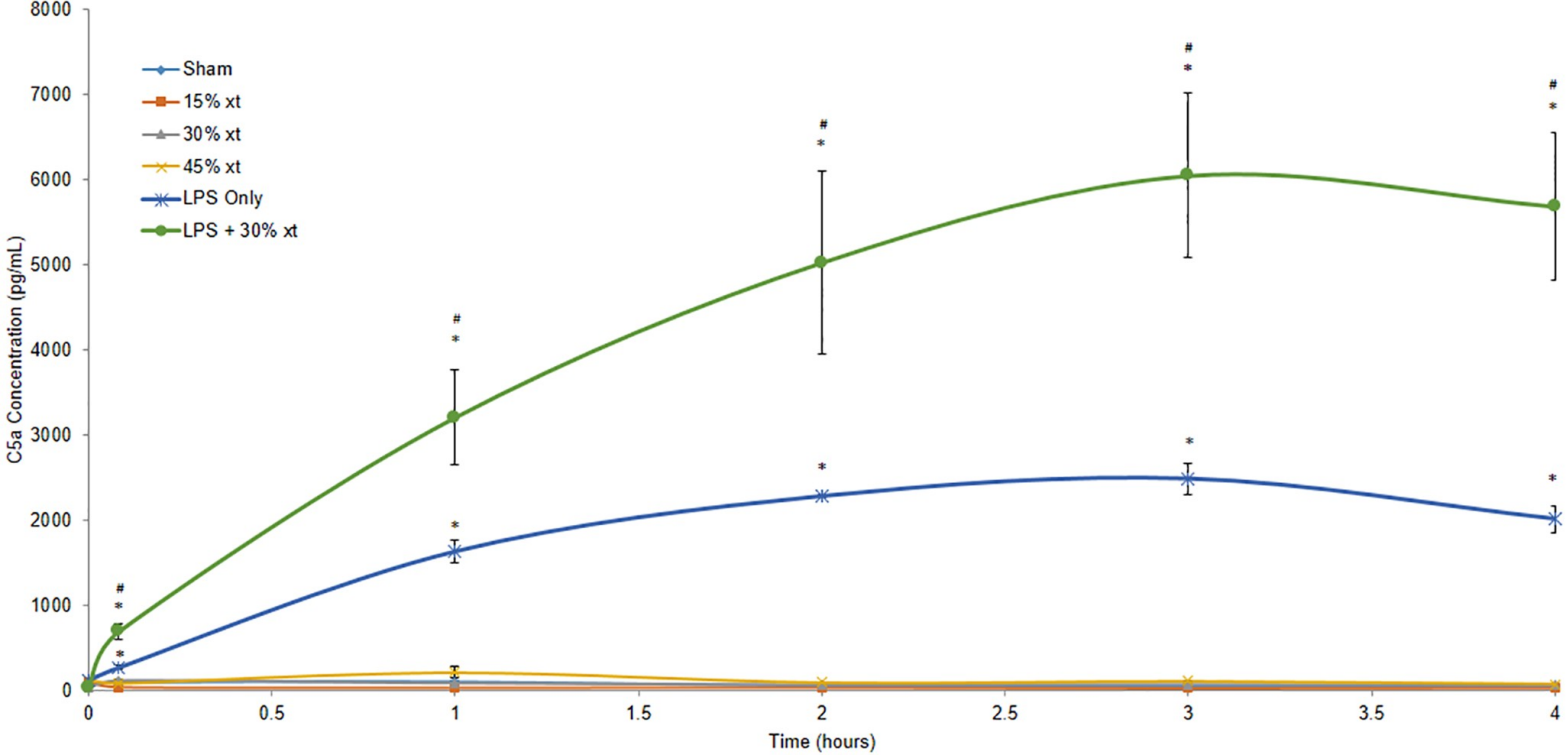
LPS alone and LPS+30% transfusion induces complement activation. Plasma was isolated from animals prior to receiving LPS alone (n = 3), 15% (n = 3), 30% (n = 3), 45% (n = 3) erythrocyte transfusion (xt) alone or LPS+30% erythrocyte transfusion (n = 3) and then at 5, 60, 120, 180 and 240 minutes after transfusion. C5a was then measured in each sample by ELISA and absorbance was read at 450 nm. Two replicates for each animal were measured for every time point. Data are means and standard error of the mean. Statistical analysis was performed using an ANOVA followed by Student *t*-test. * denotes *P* < 0.001 compared to the sham at the corresponding timepoint. # denotes *P* < 0.05 compared to LPS only at the corresponding timepoint.

LPS+30% erythrocyte transfusion resulted in a severe neutrophil mediated ALI phenotype. To ascertain if complement activation plays a role in this process, we analyzed C5a levels from rat plasma prior to animals receiving LPS alone, 15–45% erythrocyte transfusion alone or LPS+30% erythrocyte transfusion and then at 5, 60, 120, 180 and 240 minutes after transfusion. Sham animals as well as animals receiving 15–45% erythrocyte transfusion alone demonstrated baseline levels of C5a signal over the course of 4 hours (Fig 7). As previously reported, transfusion of incompatible erythrocytes into rats will induce robust classical pathway complement activation leading to hemolysis [8,9], however only trace amounts of C5a is detected presumably because the self-amplification loop of the alternative pathway required for robust C5a generation is not activated in this system. With the exception of time 0, rats treated with LPS alone showed a significant increase in C5a levels over sham animals at all other time points (5–240 minutes) (P ≤ 0.001). The generation of C5a by LPS is consistent with LPS-mediated complement activation via the alternative pathway as previously described [15–18]. Animals receiving LPS+30% erythrocyte transfusion showed significantly enhanced C5a accumulation over sham animals (P < 0.001) and animals treated with LPS only at time points 5–240 minutes (P ≤ 0.042). These findings suggest that complement activation may play a role in LPS induced ALI and that erythrocyte transfusion after LPS infusion significantly increases complement activation via the classical and alternative pathways to induce profound neutrophil-mediated ALI as shown by histology (Fig 4F).

### LPS and 30% erythrocyte transfusion results in free DNA accumulation in the blood

**Fig 8:**
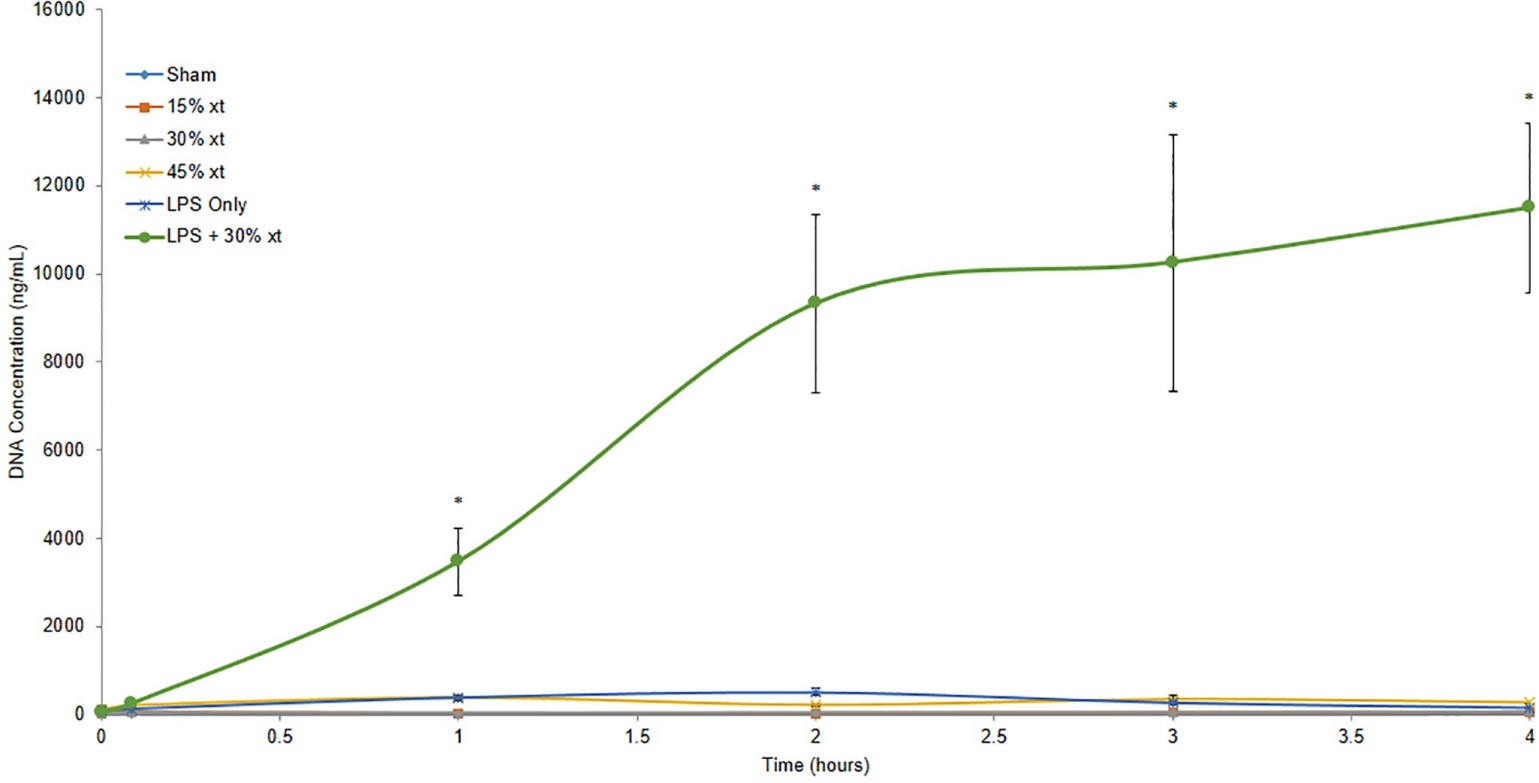
LPS+30% transfusion increases the level of free DNA in circulation. Plasma was isolated from animals prior to receiving LPS alone (n = 3), 15% (n = 3), 30% (n = 3), 45% (n = 3) erythrocyte transfusion (xt) alone or LPS+30% erythrocyte transfusion (n = 3) and then at 5, 60, 120, 180 and 240 minutes after transfusion. Plasma samples were incubated with PicoGreen. Fluorescence was read at an excitation wavelength of 485 nm and an emission wavelength of 520nm in a microplate reader. All free DNA measurements for each animal were done in triplicate. Data are means and standard error of the mean. Statistical analysis was performed using an ANOVA followed by Student *t*-test. * denotes *P* < 0.001 compared to all other experimental groups at the corresponding timepoint.

It has been previously reported in murine models of ALI that activated neutrophils release neutrophil extracellular traps (NETs) contributing to ALI. The NET biomarker free DNA is elevated in the blood of human patients with transfusion related acute lung injury (TRALI) disease [19,20]. To ascertain whether free DNA in circulation was observed in this model, DNA levels in plasma from the different treatment groups were quantified in a PicoGreen assay prior to transfusion (time 0) and at 5, 60, 120, 180 and 240 minutes after transfusion. Compared to sham, LPS only, or animals receiving 15, 30 and 45% erythrocyte transfusion, rats receiving LPS+30% transfusion had significant increases in free DNA levels at 60–240 minutes compared to all other groups (P ≤ 0.001) with a >40-fold increased level of free DNA in plasma at 4 hours for all groups (Fig 8). Taken together, the observed leukopenia, histology, increases in C5a and free DNA in circulation demonstrate that this LPS-initiated transfusion model results in severe neutrophil-mediated ALI.

## Discussion

The objective of this study was to assess if incompatible erythrocyte transfusion could induce ALI adapting our previously published rat model of antibody-initiated, complement-mediated AHTR [8]. As we have previously reported in this model, Wistar rats possessing preexisting antibodies to the A antigen of human erythrocytes initiate classical complement activation leading to a vigorous intravascular hemolysis after transfusion of type A or type AB human erythrocytes [8]. Given the inflammatory nature of the intravascular hemolysis observed in this model, we tested whether increasing the percentage of transfused human erythrocytes would induce ALI. Transfusion of 15–45% human erythrocytes alone were not sufficient to cause a significant ALI-like phenotype. It has been reported in the literature that LPS is commonly used as a ‘first-hit’ to induce ALI in ‘two-hit’ rat, mouse, sheep and swine models of ALI (reviewed in references [13,14]). To this end, we evaluated whether animals treated with LPS alone or LPS + 30% erythrocyte transfusion would elicit ALI. A 30% erythrocyte transfusion in this model is equivalent to 3 units of packed erythrocytes, for a 70 kg adult, where each unit replaces approximately the amount of erythrocytes in 500 ml of blood. In critical care/emergency medicine it is common for trauma patients (car crash, gunshot wounds, etc.) to receive multiple units of packed erythrocytes to replace lost blood. The amount of packed erythrocytes transfused can vary significantly but often exceeds the equivalent of the 30% transfusion used in our model. However, the utilization of such a high percentage of incompatible erythrocytes would be rare in clinical practice.

Animals treated with LPS alone or LPS+30% erythrocyte transfusion caused significant reduction of WBC populations at 4 hours post-transfusion suggestive of neutrophils being recruited out of circulation. Transient leukopenia is a feature of TRALI disease in humans [21]. When wet lung tissue weight was assessed, animals treated with LPS alone had a similar increase in weight compared to animals receiving LPS+30% erythrocyte transfusion. However, analysis of dry to wet lung weight ratio, as a means to quantitate changes in cellular mass, showed a significant increase in weight of the lungs for animal receiving LPS+30% erythrocyte transfusion but not LPS treated animals, which were similar in lung weight to sham treated animals (Fig 6B). The increase in lung weight ratio in the LPS+30% erythrocyte transfusion is consistent with histological findings of influx of neutrophils into the lung parenchyma (Figs 4 & 5). While the increase in wet lung weight could also be attributed to pulmonary edema, we did not measure protein leakage from the lung tissue, so the role of hydrostatic pulmonary edema in this model of ALI cannot be conclusively determined.

To further evaluate the mechanism by which LPS+30% erythrocyte transfusion could mediate the increased lung weight and ALI observed in these animals, levels of complement factor C5a in the blood were measured during the course of the four hour experiment. While animals receiving 15–45% erythrocyte transfusion alone did not generate significant levels of C5a, animals receiving LPS alone showed a significant increase in C5a generation over time. This can be attributed to the activation of the alternative pathway of complement by direct recognition of LPS by constitutively activated C3 [15–18]. Animals receiving LPS+30% erythrocyte transfusion had a significantly greater level of C5a generation that the LPS alone group (Fig 7). Our previously findings of the AIHTR model have demonstrated that transfusion of 15% mismatch erythrocytes into Wistar rats results in vigorous classical (antibody-mediated) complement pathway activation and hemolysis [8]. Thus, the significant generation of C5a in animals receiving LPS+30% erythrocyte transfusion suggests that there is a synergistic effect of LPS and erythrocyte transfusion generating a robust complement response that drives neutrophil-mediated ALI. Previous studies have implicated a role of complement in other murine models of TRALI [10,11, 22]

Given the prominent role of neutrophils in the generation of ALI in this animal model, we assessed whether free DNA would be detectable in the blood of the various treatment groups. Free DNA is considered a biomarker for neutrophil extracellular traps (NETs) and has been demonstrated to be generated in murine models of ALI and elevated levels of free DNA have been reported in the blood of human TRALI patients [19,20]. Sham animals, animals receiving erythrocyte transfusion alone (15–45%) and animals treated with LPS alone demonstrated baseline levels of free DNA over the four hour time course. In contrast, animals receiving LPS+30% erythrocyte transfusion generated significantly increased levels of free DNA (Fig 8). The levels of free DNA approach 12 μg/ml which, to our knowledge, is significantly more than what has previously been observed in rodent models of TRALI [19,20]. We attribute this robust level of free DNA to the massive neutrophil infiltration of lung parenchyma that occurs in this model. The lack of free DNA observed in animals treated with LPS only suggests that while the level of complement activation as measured by C5a in the circulation was significantly elevated, it was not sufficient to fully activate the neutrophils to undergo NETosis. We thus speculate that the combined stimulation of the classical and alternative pathways of complement by erythrocyte transfusion and LPS, respectively, lead to the generation of significant levels of C5a which recruit and activate neutrophils to mediate robust free DNA generation indicative of NETosis.

A number of ‘two-hit’ ALI models, utilizing LPS as the ‘first-hit’ and either an antibody-dependent (e.g. H2K^d^, HLA, etc.) or an antibody-independent (e.g. aged RBCs, lysoPCs, RBC supernatants, etc.) stimulus as the ‘second hit’ have been published in the literature in a variety of species (reviewed in references [23,24]). To our knowledge our ALI model is unique in that it uses transfused incompatible allogeneic erythrocytes as the ‘second hit’ while the few other in vivo models reported in the literature that transfuse erythrocytes utilize syngeneic erythrocytes [25–28]. AHTRs following erythrocyte transfusions in humans occur with incompatible allogeneic erythrocyte transfusions. This new model provides a robust ALI phenotype after LPS and incompatible allogeneic erythrocyte transfusion, which is mediated by complement-induced neutrophil lung infiltration. We believe this will be a useful model for refining our understanding of complement and neutrophil-mediated acute lung injury pathogenesis.

## Supporting information

S1 File ANOVA Post Hoc Tests. **Results of the ANOVA post hoc analysis**. Excel spreadsheet containing minimal data sets for Figs 2–8.
(DOCX)
