# Supplementary material for: Incompatible erythrocyte transfusion with lipopolysaccharide induces acute lung injury in a novel rat model
Source: PLoS One. 2020 Apr 20;15(4):e0230482. doi: 10.1371/journal.pone.0230482 (PMC7170260; doi:10.1371/journal.pone.0230482)
Supplement: S1 File — Results of the ANOVA post hoc analysis. Excel spreadsheet containing minimal data sets for Figs 2–8. (DOCX) [file pone.0230482.s001.docx]

At 5 minutes post-transfusion, white blood cells (WBCs) were at levels similar to time 0 and sham-treated animals (P=0.96). **(Fig 3A)**

| **Descriptives** | | | | | | | | |
| --- | --- | --- | --- | --- | --- | --- | --- | --- |
| WBC | | | | | | | | |
|  | N | Mean | Std. Deviation | Std. Error | 95% Confidence Interval for Mean | | Minimum | Maximum |
|  |  |  |  |  | Lower Bound | Upper Bound |  |  |
| Pre | 64 | 5.7547 | 1.96775 | .24597 | 5.2632 | 6.2462 | 2.00 | 10.30 |
| Sham | 5 | 5.2000 | 1.28452 | .57446 | 3.6051 | 6.7949 | 3.30 | 6.70 |
| 15% | 3 | 5.6667 | 1.55349 | .89691 | 1.8076 | 9.5258 | 4.40 | 7.40 |
| 30% | 3 | 5.9667 | .40415 | .23333 | 4.9627 | 6.9706 | 5.60 | 6.40 |
| 45% | 3 | 5.2333 | .49329 | .28480 | 4.0079 | 6.4587 | 4.90 | 5.80 |
| Total | 78 | 5.7038 | 1.83221 | .20746 | 5.2907 | 6.1169 | 2.00 | 10.30 |

| **Test of Homogeneity of Variances** | | | | | |
| --- | --- | --- | --- | --- | --- |
|  | | Levene Statistic | df1 | df2 | Sig. |
| WBC | Based on Mean | 2.085 | 4 | 73 | .091 |
|  | Based on Median | 2.186 | 4 | 73 | .079 |
|  | Based on Median and with adjusted df | 2.186 | 4 | 68.591 | .080 |
|  | Based on trimmed mean | 2.073 | 4 | 73 | .093 |

| **ANOVA** | | | | | |
| --- | --- | --- | --- | --- | --- |
| WBC | | | | | |
|  | Sum of Squares | df | Mean Square | F | Sig. |
| Between Groups | 2.310 | 4 | .578 | .165 | .956 |
| Within Groups | 256.179 | 73 | 3.509 |  |  |
| Total | 258.489 | 77 |  |  |  |

| **Robust Tests of Equality of Means** | | | | |
| --- | --- | --- | --- | --- |
| WBC | | | | |
|  | Statistic^a^ | df1 | df2 | Sig. |
| Welch | .914 | 4 | 6.660 | .508 |
| a. Asymptotically F distributed. | | | | |

| **Multiple Comparisons** | | | | | | | |
| --- | --- | --- | --- | --- | --- | --- | --- |
| Dependent Variable: WBC | | | | | | | |
|  | (I) Class1 | (J) Class1 | Mean Difference (I-J) | Std. Error | Sig. | 95% Confidence Interval | |
|  |  |  |  |  |  | Lower Bound | Upper Bound |
| Hochberg | Pre | Sham | .55469 | .86988 | .999 | -1.9523 | 3.0616 |
|  |  | 15% | .08802 | 1.10662 | 1.000 | -3.1012 | 3.2772 |
|  |  | 30% | -.21198 | 1.10662 | 1.000 | -3.4012 | 2.9772 |
|  |  | 45% | .52135 | 1.10662 | 1.000 | -2.6679 | 3.7106 |
|  | Sham | Pre | -.55469 | .86988 | .999 | -3.0616 | 1.9523 |
|  |  | 15% | -.46667 | 1.36807 | 1.000 | -4.4094 | 3.4761 |
|  |  | 30% | -.76667 | 1.36807 | 1.000 | -4.7094 | 3.1761 |
|  |  | 45% | -.03333 | 1.36807 | 1.000 | -3.9761 | 3.9094 |
|  | 15% | Pre | -.08802 | 1.10662 | 1.000 | -3.2772 | 3.1012 |
|  |  | Sham | .46667 | 1.36807 | 1.000 | -3.4761 | 4.4094 |
|  |  | 30% | -.30000 | 1.52955 | 1.000 | -4.7081 | 4.1081 |
|  |  | 45% | .43333 | 1.52955 | 1.000 | -3.9748 | 4.8414 |
|  | 30% | Pre | .21198 | 1.10662 | 1.000 | -2.9772 | 3.4012 |
|  |  | Sham | .76667 | 1.36807 | 1.000 | -3.1761 | 4.7094 |
|  |  | 15% | .30000 | 1.52955 | 1.000 | -4.1081 | 4.7081 |
|  |  | 45% | .73333 | 1.52955 | 1.000 | -3.6748 | 5.1414 |
|  | 45% | Pre | -.52135 | 1.10662 | 1.000 | -3.7106 | 2.6679 |
|  |  | Sham | .03333 | 1.36807 | 1.000 | -3.9094 | 3.9761 |
|  |  | 15% | -.43333 | 1.52955 | 1.000 | -4.8414 | 3.9748 |
|  |  | 30% | -.73333 | 1.52955 | 1.000 | -5.1414 | 3.6748 |
| Dunnett t (2-sided)^a^ | Sham | Pre | -.55469 | .86988 | .948 | -2.7734 | 1.6640 |
|  | 15% | Pre | -.08802 | 1.10662 | 1.000 | -2.9106 | 2.7345 |
|  | 30% | Pre | .21198 | 1.10662 | .999 | -2.6106 | 3.0345 |
|  | 45% | Pre | -.52135 | 1.10662 | .982 | -3.3439 | 2.3012 |
| a. Dunnett t-tests treat one group as a control, and compare all other groups against it. | | | | | | | |

At four hours post-transfusion, WBC levels were slightly increased for animals receiving 15 and 30% erythrocyte transfusion whereas animals that received the 45% transfusion were significantly elevated compared to time 0 (P<=0.001) **(Fig 3A)**

| **Descriptives** | | | | | | | | |
| --- | --- | --- | --- | --- | --- | --- | --- | --- |
| WBC | | | | | | | | |
|  | N | Mean | Std. Deviation | Std. Error | 95% Confidence Interval for Mean | | Minimum | Maximum |
|  |  |  |  |  | Lower Bound | Upper Bound |  |  |
| Pre | 64 | 5.7547 | 1.96775 | .24597 | 5.2632 | 6.2462 | 2.00 | 10.30 |
| Sham | 8 | 4.8250 | 1.35515 | .47912 | 3.6921 | 5.9579 | 3.10 | 7.60 |
| 15% | 6 | 6.5667 | 1.25645 | .51294 | 5.2481 | 7.8852 | 4.80 | 8.50 |
| 30% | 9 | 6.7556 | 2.24839 | .74946 | 5.0273 | 8.4838 | 1.80 | 9.90 |
| 45% | 6 | 9.9000 | 3.47620 | 1.41915 | 6.2519 | 13.5481 | 5.50 | 13.50 |
| Total | 93 | 6.0914 | 2.27938 | .23636 | 5.6220 | 6.5608 | 1.80 | 13.50 |

| **Test of Homogeneity of Variances** | | | | | |
| --- | --- | --- | --- | --- | --- |
|  | | Levene Statistic | df1 | df2 | Sig. |
| WBC | Based on Mean | 3.552 | 4 | 88 | .010 |
|  | Based on Median | 3.495 | 4 | 88 | .011 |
|  | Based on Median and with adjusted df | 3.495 | 4 | 78.039 | .011 |
|  | Based on trimmed mean | 3.543 | 4 | 88 | .010 |

| **ANOVA** | | | | | |
| --- | --- | --- | --- | --- | --- |
| WBC | | | | | |
|  | Sum of Squares | df | Mean Square | F | Sig. |
| Between Groups | 112.444 | 4 | 28.111 | 6.767 | .000 |
| Within Groups | 365.549 | 88 | 4.154 |  |  |
| Total | 477.993 | 92 |  |  |  |

| **Robust Tests of Equality of Means** | | | | |
| --- | --- | --- | --- | --- |
| WBC | | | | |
|  | Statistic^a^ | df1 | df2 | Sig. |
| Welch | 3.576 | 4 | 14.489 | .032 |
| a. Asymptotically F distributed. | | | | |

| **Multiple Comparisons** | | | | | | | |
| --- | --- | --- | --- | --- | --- | --- | --- |
| Dependent Variable: WBC | | | | | | | |
|  | (I) Class1 | (J) Class1 | Mean Difference (I-J) | Std. Error | Sig. | 95% Confidence Interval | |
|  |  |  |  |  |  | Lower Bound | Upper Bound |
| Bonferroni | Pre | Sham | .92969 | .76430 | 1.000 | -1.2711 | 3.1305 |
|  |  | 15% | -.81198 | .87019 | 1.000 | -3.3177 | 1.6938 |
|  |  | 30% | -1.00087 | .72557 | 1.000 | -3.0902 | 1.0884 |
|  |  | 45% | -4.14531^*^ | .87019 | .000 | -6.6511 | -1.6396 |
|  | Sham | Pre | -.92969 | .76430 | 1.000 | -3.1305 | 1.2711 |
|  |  | 15% | -1.74167 | 1.10072 | 1.000 | -4.9112 | 1.4279 |
|  |  | 30% | -1.93056 | .99035 | .544 | -4.7823 | .9212 |
|  |  | 45% | -5.07500^*^ | 1.10072 | .000 | -8.2445 | -1.9055 |
|  | 15% | Pre | .81198 | .87019 | 1.000 | -1.6938 | 3.3177 |
|  |  | Sham | 1.74167 | 1.10072 | 1.000 | -1.4279 | 4.9112 |
|  |  | 30% | -.18889 | 1.07419 | 1.000 | -3.2820 | 2.9043 |
|  |  | 45% | -3.33333 | 1.17671 | .057 | -6.7217 | .0551 |
|  | 30% | Pre | 1.00087 | .72557 | 1.000 | -1.0884 | 3.0902 |
|  |  | Sham | 1.93056 | .99035 | .544 | -.9212 | 4.7823 |
|  |  | 15% | .18889 | 1.07419 | 1.000 | -2.9043 | 3.2820 |
|  |  | 45% | -3.14444^*^ | 1.07419 | .044 | -6.2376 | -.0513 |
|  | 45% | Pre | 4.14531^*^ | .87019 | .000 | 1.6396 | 6.6511 |
|  |  | Sham | 5.07500^*^ | 1.10072 | .000 | 1.9055 | 8.2445 |
|  |  | 15% | 3.33333 | 1.17671 | .057 | -.0551 | 6.7217 |
|  |  | 30% | 3.14444^*^ | 1.07419 | .044 | .0513 | 6.2376 |
| Hochberg | Pre | Sham | .92969 | .76430 | .917 | -1.2621 | 3.1215 |
|  |  | 15% | -.81198 | .87019 | .985 | -3.3074 | 1.6835 |
|  |  | 30% | -1.00087 | .72557 | .837 | -3.0816 | 1.0799 |
|  |  | 45% | -4.14531^*^ | .87019 | .000 | -6.6408 | -1.6498 |
|  | Sham | Pre | -.92969 | .76430 | .917 | -3.1215 | 1.2621 |
|  |  | 15% | -1.74167 | 1.10072 | .700 | -4.8982 | 1.4149 |
|  |  | 30% | -1.93056 | .99035 | .419 | -4.7706 | .9095 |
|  |  | 45% | -5.07500^*^ | 1.10072 | .000 | -8.2315 | -1.9185 |
|  | 15% | Pre | .81198 | .87019 | .985 | -1.6835 | 3.3074 |
|  |  | Sham | 1.74167 | 1.10072 | .700 | -1.4149 | 4.8982 |
|  |  | 30% | -.18889 | 1.07419 | 1.000 | -3.2694 | 2.8916 |
|  |  | 45% | -3.33333 | 1.17671 | .055 | -6.7078 | .0412 |
|  | 30% | Pre | 1.00087 | .72557 | .837 | -1.0799 | 3.0816 |
|  |  | Sham | 1.93056 | .99035 | .419 | -.9095 | 4.7706 |
|  |  | 15% | .18889 | 1.07419 | 1.000 | -2.8916 | 3.2694 |
|  |  | 45% | -3.14444^*^ | 1.07419 | .042 | -6.2249 | -.0640 |
|  | 45% | Pre | 4.14531^*^ | .87019 | .000 | 1.6498 | 6.6408 |
|  |  | Sham | 5.07500^*^ | 1.10072 | .000 | 1.9185 | 8.2315 |
|  |  | 15% | 3.33333 | 1.17671 | .055 | -.0412 | 6.7078 |
|  |  | 30% | 3.14444^*^ | 1.07419 | .042 | .0640 | 6.2249 |
| Dunnett t (2-sided)^b^ | Sham | Pre | -.92969 | .76430 | .635 | -2.8701 | 1.0107 |
|  | 15% | Pre | .81198 | .87019 | .819 | -1.3972 | 3.0212 |
|  | 30% | Pre | 1.00087 | .72557 | .520 | -.8412 | 2.8429 |
|  | 45% | Pre | 4.14531^*^ | .87019 | .000 | 1.9361 | 6.3545 |
| *. The mean difference is significant at the 0.05 level. | | | | | | | |
| b. Dunnett t-tests treat one group as a control, and compare all other groups against it. | | | | | | | |

A 2 mg/kg LPS intravenous infusion given alone (P=0.005) or followed by 30% erythrocyte transfusion (P<0.001) resulted in a marked increase in blood levels of WBCs at 5 minutes compared to time 0 controls **(Fig 3A)**

| **Multiple Comparisons** | | | | | | | |
| --- | --- | --- | --- | --- | --- | --- | --- |
| Dependent Variable: WBC | | | | | | | |
|  | (I) Class | (J) Class | Mean Difference (I-J) | Std. Error | Sig. | 95% Confidence Interval | |
|  |  |  |  |  |  | Lower Bound | Upper Bound |
| Hochberg | Pre | Sham | .55469 | .85468 | 1.000 | -2.1174 | 3.2268 |
|  |  | 15% | .08802 | 1.08728 | 1.000 | -3.3113 | 3.4873 |
|  |  | 30% | -.21198 | 1.08728 | 1.000 | -3.6113 | 3.1873 |
|  |  | 45% | .52135 | 1.08728 | 1.000 | -2.8779 | 3.9206 |
|  |  | LPS Only | -3.64531^*^ | .94862 | .005 | -6.6111 | -.6796 |
|  |  | LPS + 30% | -5.01198^*^ | 1.08728 | .000 | -8.4113 | -1.6127 |
|  | Sham | Pre | -.55469 | .85468 | 1.000 | -3.2268 | 2.1174 |
|  |  | 15% | -.46667 | 1.34417 | 1.000 | -4.6691 | 3.7358 |
|  |  | 30% | -.76667 | 1.34417 | 1.000 | -4.9691 | 3.4358 |
|  |  | 45% | -.03333 | 1.34417 | 1.000 | -4.2358 | 4.1691 |
|  |  | LPS Only | -4.20000^*^ | 1.23470 | .022 | -8.0602 | -.3398 |
|  |  | LPS + 30% | -5.56667^*^ | 1.34417 | .002 | -9.7691 | -1.3642 |
|  | 15% | Pre | -.08802 | 1.08728 | 1.000 | -3.4873 | 3.3113 |
|  |  | Sham | .46667 | 1.34417 | 1.000 | -3.7358 | 4.6691 |
|  |  | 30% | -.30000 | 1.50283 | 1.000 | -4.9984 | 4.3984 |
|  |  | 45% | .43333 | 1.50283 | 1.000 | -4.2651 | 5.1318 |
|  |  | LPS Only | -3.73333 | 1.40577 | .177 | -8.1283 | .6617 |
|  |  | LPS + 30% | -5.10000^*^ | 1.50283 | .022 | -9.7984 | -.4016 |
|  | 30% | Pre | .21198 | 1.08728 | 1.000 | -3.1873 | 3.6113 |
|  |  | Sham | .76667 | 1.34417 | 1.000 | -3.4358 | 4.9691 |
|  |  | 15% | .30000 | 1.50283 | 1.000 | -4.3984 | 4.9984 |
|  |  | 45% | .73333 | 1.50283 | 1.000 | -3.9651 | 5.4318 |
|  |  | LPS Only | -3.43333 | 1.40577 | .288 | -7.8283 | .9617 |
|  |  | LPS + 30% | -4.80000^*^ | 1.50283 | .041 | -9.4984 | -.1016 |
|  | 45% | Pre | -.52135 | 1.08728 | 1.000 | -3.9206 | 2.8779 |
|  |  | Sham | .03333 | 1.34417 | 1.000 | -4.1691 | 4.2358 |
|  |  | 15% | -.43333 | 1.50283 | 1.000 | -5.1318 | 4.2651 |
|  |  | 30% | -.73333 | 1.50283 | 1.000 | -5.4318 | 3.9651 |
|  |  | LPS Only | -4.16667 | 1.40577 | .079 | -8.5617 | .2283 |
|  |  | LPS + 30% | -5.53333^*^ | 1.50283 | .009 | -10.2318 | -.8349 |
|  | LPS Only | Pre | 3.64531^*^ | .94862 | .005 | .6796 | 6.6111 |
|  |  | Sham | 4.20000^*^ | 1.23470 | .022 | .3398 | 8.0602 |
|  |  | 15% | 3.73333 | 1.40577 | .177 | -.6617 | 8.1283 |
|  |  | 30% | 3.43333 | 1.40577 | .288 | -.9617 | 7.8283 |
|  |  | 45% | 4.16667 | 1.40577 | .079 | -.2283 | 8.5617 |
|  |  | LPS + 30% | -1.36667 | 1.40577 | 1.000 | -5.7617 | 3.0283 |
|  | LPS + 30% | Pre | 5.01198^*^ | 1.08728 | .000 | 1.6127 | 8.4113 |
|  |  | Sham | 5.56667^*^ | 1.34417 | .002 | 1.3642 | 9.7691 |
|  |  | 15% | 5.10000^*^ | 1.50283 | .022 | .4016 | 9.7984 |
|  |  | 30% | 4.80000^*^ | 1.50283 | .041 | .1016 | 9.4984 |
|  |  | 45% | 5.53333^*^ | 1.50283 | .009 | .8349 | 10.2318 |
|  |  | LPS Only | 1.36667 | 1.40577 | 1.000 | -3.0283 | 5.7617 |
| Dunnett t (2-sided)^b^ | Sham | Pre | -.55469 | .85468 | .986 | -2.8584 | 1.7491 |
|  | 15% | Pre | -.08802 | 1.08728 | 1.000 | -3.0187 | 2.8427 |
|  | 30% | Pre | .21198 | 1.08728 | 1.000 | -2.7187 | 3.1427 |
|  | 45% | Pre | -.52135 | 1.08728 | .997 | -3.4521 | 2.4094 |
|  | LPS Only | Pre | 3.64531^*^ | .94862 | .001 | 1.0884 | 6.2023 |
|  | LPS + 30% | Pre | 5.01198^*^ | 1.08728 | .000 | 2.0813 | 7.9427 |
| *. The mean difference is significant at the 0.05 level. | | | | | | | |
| b. Dunnett t-tests treat one group as a control, and compare all other groups against it. | | | | | | | |

In contrast, a significant reduction in circulating levels of WBC was observed at 4 hours compared to pre-transfusion controls for both LPS only (P<0.001) and LPS+30% erythrocyte transfusion (P<0.001) **(Fig 3A)**

| **Multiple Comparisons** | | | | | | | |
| --- | --- | --- | --- | --- | --- | --- | --- |
| Dependent Variable: WBC | | | | | | | |
|  | (I) Class | (J) Class | Mean Difference (I-J) | Std. Error | Sig. | 95% Confidence Interval | |
|  |  |  |  |  |  | Lower Bound | Upper Bound |
| Bonferroni | Pre | Sham | .92969 | .72152 | 1.000 | -1.3144 | 3.1738 |
|  |  | 15% | -.81198 | .82149 | 1.000 | -3.3670 | 1.7431 |
|  |  | 30% | -1.00087 | .68497 | 1.000 | -3.1313 | 1.1295 |
|  |  | 45% | -4.14531^*^ | .82149 | .000 | -6.7003 | -1.5903 |
|  |  | LPS Only | 2.58196^*^ | .62800 | .000 | .6287 | 4.5352 |
|  |  | LPS + 30% | 2.64700^*^ | .58533 | .000 | .8265 | 4.4675 |
|  | Sham | Pre | -.92969 | .72152 | 1.000 | -3.1738 | 1.3144 |
|  |  | 15% | -1.74167 | 1.03911 | 1.000 | -4.9736 | 1.4902 |
|  |  | 30% | -1.93056 | .93492 | .867 | -4.8384 | .9773 |
|  |  | 45% | -5.07500^*^ | 1.03911 | .000 | -8.3069 | -1.8431 |
|  |  | LPS Only | 1.65227 | .89403 | 1.000 | -1.1284 | 4.4329 |
|  |  | LPS + 30% | 1.71731 | .86459 | 1.000 | -.9718 | 4.4064 |
|  | 15% | Pre | .81198 | .82149 | 1.000 | -1.7431 | 3.3670 |
|  |  | Sham | 1.74167 | 1.03911 | 1.000 | -1.4902 | 4.9736 |
|  |  | 30% | -.18889 | 1.01407 | 1.000 | -3.3429 | 2.9651 |
|  |  | 45% | -3.33333 | 1.11086 | .070 | -6.7884 | .1217 |
|  |  | LPS Only | 3.39394^*^ | .97650 | .015 | .3568 | 6.4311 |
|  |  | LPS + 30% | 3.45897^*^ | .94962 | .009 | .5054 | 6.4125 |
|  | 30% | Pre | 1.00087 | .68497 | 1.000 | -1.1295 | 3.1313 |
|  |  | Sham | 1.93056 | .93492 | .867 | -.9773 | 4.8384 |
|  |  | 15% | .18889 | 1.01407 | 1.000 | -2.9651 | 3.3429 |
|  |  | 45% | -3.14444 | 1.01407 | .051 | -6.2984 | .0096 |
|  |  | LPS Only | 3.58283^*^ | .86480 | .001 | .8931 | 6.2726 |
|  |  | LPS + 30% | 3.64786^*^ | .83433 | .001 | 1.0529 | 6.2428 |
|  | 45% | Pre | 4.14531^*^ | .82149 | .000 | 1.5903 | 6.7003 |
|  |  | Sham | 5.07500^*^ | 1.03911 | .000 | 1.8431 | 8.3069 |
|  |  | 15% | 3.33333 | 1.11086 | .070 | -.1217 | 6.7884 |
|  |  | 30% | 3.14444 | 1.01407 | .051 | -.0096 | 6.2984 |
|  |  | LPS Only | 6.72727^*^ | .97650 | .000 | 3.6901 | 9.7644 |
|  |  | LPS + 30% | 6.79231^*^ | .94962 | .000 | 3.8388 | 9.7458 |
|  | LPS Only | Pre | -2.58196^*^ | .62800 | .000 | -4.5352 | -.6287 |
|  |  | Sham | -1.65227 | .89403 | 1.000 | -4.4329 | 1.1284 |
|  |  | 15% | -3.39394^*^ | .97650 | .015 | -6.4311 | -.3568 |
|  |  | 30% | -3.58283^*^ | .86480 | .001 | -6.2726 | -.8931 |
|  |  | 45% | -6.72727^*^ | .97650 | .000 | -9.7644 | -3.6901 |
|  |  | LPS + 30% | .06503 | .78824 | 1.000 | -2.3866 | 2.5166 |
|  | LPS + 30% | Pre | -2.64700^*^ | .58533 | .000 | -4.4675 | -.8265 |
|  |  | Sham | -1.71731 | .86459 | 1.000 | -4.4064 | .9718 |
|  |  | 15% | -3.45897^*^ | .94962 | .009 | -6.4125 | -.5054 |
|  |  | 30% | -3.64786^*^ | .83433 | .001 | -6.2428 | -1.0529 |
|  |  | 45% | -6.79231^*^ | .94962 | .000 | -9.7458 | -3.8388 |
|  |  | LPS Only | -.06503 | .78824 | 1.000 | -2.5166 | 2.3866 |
| Hochberg | Pre | Sham | .92969 | .72152 | .988 | -1.3058 | 3.1652 |
|  |  | 15% | -.81198 | .82149 | 1.000 | -3.3572 | 1.7332 |
|  |  | 30% | -1.00087 | .68497 | .957 | -3.1231 | 1.1213 |
|  |  | 45% | -4.14531^*^ | .82149 | .000 | -6.6905 | -1.6001 |
|  |  | LPS Only | 2.58196^*^ | .62800 | .000 | .6362 | 4.5277 |
|  |  | LPS + 30% | 2.64700^*^ | .58533 | .000 | .8335 | 4.4605 |
|  | Sham | Pre | -.92969 | .72152 | .988 | -3.1652 | 1.3058 |
|  |  | 15% | -1.74167 | 1.03911 | .866 | -4.9611 | 1.4778 |
|  |  | 30% | -1.93056 | .93492 | .570 | -4.8272 | .9661 |
|  |  | 45% | -5.07500^*^ | 1.03911 | .000 | -8.2944 | -1.8556 |
|  |  | LPS Only | 1.65227 | .89403 | .750 | -1.1177 | 4.4222 |
|  |  | LPS + 30% | 1.71731 | .86459 | .637 | -.9614 | 4.3960 |
|  | 15% | Pre | .81198 | .82149 | 1.000 | -1.7332 | 3.3572 |
|  |  | Sham | 1.74167 | 1.03911 | .866 | -1.4778 | 4.9611 |
|  |  | 30% | -.18889 | 1.01407 | 1.000 | -3.3307 | 2.9530 |
|  |  | 45% | -3.33333 | 1.11086 | .067 | -6.7751 | .1084 |
|  |  | LPS Only | 3.39394^*^ | .97650 | .015 | .3685 | 6.4194 |
|  |  | LPS + 30% | 3.45897^*^ | .94962 | .009 | .5168 | 6.4011 |
|  | 30% | Pre | 1.00087 | .68497 | .957 | -1.1213 | 3.1231 |
|  |  | Sham | 1.93056 | .93492 | .570 | -.9661 | 4.8272 |
|  |  | 15% | .18889 | 1.01407 | 1.000 | -2.9530 | 3.3307 |
|  |  | 45% | -3.14444^*^ | 1.01407 | .050 | -6.2863 | -.0026 |
|  |  | LPS Only | 3.58283^*^ | .86480 | .001 | .9034 | 6.2622 |
|  |  | LPS + 30% | 3.64786^*^ | .83433 | .001 | 1.0629 | 6.2328 |
|  | 45% | Pre | 4.14531^*^ | .82149 | .000 | 1.6001 | 6.6905 |
|  |  | Sham | 5.07500^*^ | 1.03911 | .000 | 1.8556 | 8.2944 |
|  |  | 15% | 3.33333 | 1.11086 | .067 | -.1084 | 6.7751 |
|  |  | 30% | 3.14444^*^ | 1.01407 | .050 | .0026 | 6.2863 |
|  |  | LPS Only | 6.72727^*^ | .97650 | .000 | 3.7018 | 9.7527 |
|  |  | LPS + 30% | 6.79231^*^ | .94962 | .000 | 3.8501 | 9.7345 |
|  | LPS Only | Pre | -2.58196^*^ | .62800 | .000 | -4.5277 | -.6362 |
|  |  | Sham | -1.65227 | .89403 | .750 | -4.4222 | 1.1177 |
|  |  | 15% | -3.39394^*^ | .97650 | .015 | -6.4194 | -.3685 |
|  |  | 30% | -3.58283^*^ | .86480 | .001 | -6.2622 | -.9034 |
|  |  | 45% | -6.72727^*^ | .97650 | .000 | -9.7527 | -3.7018 |
|  |  | LPS + 30% | .06503 | .78824 | 1.000 | -2.3771 | 2.5072 |
|  | LPS + 30% | Pre | -2.64700^*^ | .58533 | .000 | -4.4605 | -.8335 |
|  |  | Sham | -1.71731 | .86459 | .637 | -4.3960 | .9614 |
|  |  | 15% | -3.45897^*^ | .94962 | .009 | -6.4011 | -.5168 |
|  |  | 30% | -3.64786^*^ | .83433 | .001 | -6.2328 | -1.0629 |
|  |  | 45% | -6.79231^*^ | .94962 | .000 | -9.7345 | -3.8501 |
|  |  | LPS Only | -.06503 | .78824 | 1.000 | -2.5072 | 2.3771 |
| Dunnett t (2-sided)^b^ | Sham | Pre | -.92969 | .72152 | .725 | -2.8586 | .9993 |
|  | 15% | Pre | .81198 | .82149 | .898 | -1.3842 | 3.0082 |
|  | 30% | Pre | 1.00087 | .68497 | .600 | -.8303 | 2.8321 |
|  | 45% | Pre | 4.14531^*^ | .82149 | .000 | 1.9491 | 6.3415 |
|  | LPS Only | Pre | -2.58196^*^ | .62800 | .000 | -4.2609 | -.9030 |
|  | LPS + 30% | Pre | -2.64700^*^ | .58533 | .000 | -4.2118 | -1.0822 |
| *. The mean difference is significant at the 0.05 level. | | | | | | | |
| b. Dunnett t-tests treat one group as a control, and compare all other groups against it. | | | | | | | |

The increase in WBCs at 5 min and reduction at 4 hours was also significant when comparing the 30% erythrocyte transfusion group to animal receiving LPS+30% erythrocyte transfusion (P=0.017 and P<0.001, respectively) **(Fig 3A)**

| **Multiple Comparisons** | | | | | | | |
| --- | --- | --- | --- | --- | --- | --- | --- |
| Dependent Variable: WBC | | | | | | | |
|  | (I) Class30 | (J) Class30 | Mean Difference (I-J) | Std. Error | Sig. | 95% Confidence Interval | |
|  |  |  |  |  |  | Lower Bound | Upper Bound |
| Bonferroni | Pre | 30% | -.21198 | 1.12156 | 1.000 | -3.2574 | 2.8334 |
|  |  | LPS Only | -3.64531^*^ | .97852 | .002 | -6.3023 | -.9883 |
|  |  | LPS + 30% | -5.01198^*^ | 1.12156 | .000 | -8.0574 | -1.9666 |
|  | 30% | Pre | .21198 | 1.12156 | 1.000 | -2.8334 | 3.2574 |
|  |  | LPS Only | -3.43333 | 1.45009 | .124 | -7.3708 | .5041 |
|  |  | LPS + 30% | -4.80000^*^ | 1.55021 | .017 | -9.0093 | -.5907 |
|  | LPS Only | Pre | 3.64531^*^ | .97852 | .002 | .9883 | 6.3023 |
|  |  | 30% | 3.43333 | 1.45009 | .124 | -.5041 | 7.3708 |
|  |  | LPS + 30% | -1.36667 | 1.45009 | 1.000 | -5.3041 | 2.5708 |
|  | LPS + 30% | Pre | 5.01198^*^ | 1.12156 | .000 | 1.9666 | 8.0574 |
|  |  | 30% | 4.80000^*^ | 1.55021 | .017 | .5907 | 9.0093 |
|  |  | LPS Only | 1.36667 | 1.45009 | 1.000 | -2.5708 | 5.3041 |
| Hochberg | Pre | 30% | -.21198 | 1.12156 | 1.000 | -3.2444 | 2.8204 |
|  |  | LPS Only | -3.64531^*^ | .97852 | .002 | -6.2910 | -.9997 |
|  |  | LPS + 30% | -5.01198^*^ | 1.12156 | .000 | -8.0444 | -1.9796 |
|  | 30% | Pre | .21198 | 1.12156 | 1.000 | -2.8204 | 3.2444 |
|  |  | LPS Only | -3.43333 | 1.45009 | .116 | -7.3540 | .4873 |
|  |  | LPS + 30% | -4.80000^*^ | 1.55021 | .017 | -8.9913 | -.6087 |
|  | LPS Only | Pre | 3.64531^*^ | .97852 | .002 | .9997 | 6.2910 |
|  |  | 30% | 3.43333 | 1.45009 | .116 | -.4873 | 7.3540 |
|  |  | LPS + 30% | -1.36667 | 1.45009 | .920 | -5.2873 | 2.5540 |
|  | LPS + 30% | Pre | 5.01198^*^ | 1.12156 | .000 | 1.9796 | 8.0444 |
|  |  | 30% | 4.80000^*^ | 1.55021 | .017 | .6087 | 8.9913 |
|  |  | LPS Only | 1.36667 | 1.45009 | .920 | -2.5540 | 5.2873 |
| Dunnett t (2-sided)^b^ | 30% | Pre | .21198 | 1.12156 | .997 | -2.5287 | 2.9527 |
|  | LPS Only | Pre | 3.64531^*^ | .97852 | .001 | 1.2542 | 6.0365 |
|  | LPS + 30% | Pre | 5.01198^*^ | 1.12156 | .000 | 2.2713 | 7.7527 |
| *. The mean difference is significant at the 0.05 level. | | | | | | | |
| b. Dunnett t-tests treat one group as a control, and compare all other groups against it. | | | | | | | |

| **Multiple Comparisons** | | | | | | | |
| --- | --- | --- | --- | --- | --- | --- | --- |
| Dependent Variable: WBC | | | | | | | |
|  | (I) Class30 | (J) Class30 | Mean Difference (I-J) | Std. Error | Sig. | 95% Confidence Interval | |
|  |  |  |  |  |  | Lower Bound | Upper Bound |
| Bonferroni | Pre | 30% | -1.00087 | .66658 | .820 | -2.7979 | .7961 |
|  |  | LPS Only | 2.58196^*^ | .61115 | .000 | .9344 | 4.2295 |
|  |  | LPS + 30% | 2.64700^*^ | .56962 | .000 | 1.1114 | 4.1826 |
|  | 30% | Pre | 1.00087 | .66658 | .820 | -.7961 | 2.7979 |
|  |  | LPS Only | 3.58283^*^ | .84159 | .000 | 1.3140 | 5.8516 |
|  |  | LPS + 30% | 3.64786^*^ | .81193 | .000 | 1.4590 | 5.8367 |
|  | LPS Only | Pre | -2.58196^*^ | .61115 | .000 | -4.2295 | -.9344 |
|  |  | 30% | -3.58283^*^ | .84159 | .000 | -5.8516 | -1.3140 |
|  |  | LPS + 30% | .06503 | .76708 | 1.000 | -2.0029 | 2.1330 |
|  | LPS + 30% | Pre | -2.64700^*^ | .56962 | .000 | -4.1826 | -1.1114 |
|  |  | 30% | -3.64786^*^ | .81193 | .000 | -5.8367 | -1.4590 |
|  |  | LPS Only | -.06503 | .76708 | 1.000 | -2.1330 | 2.0029 |
| Hochberg | Pre | 30% | -1.00087 | .66658 | .579 | -2.7910 | .7892 |
|  |  | LPS Only | 2.58196^*^ | .61115 | .000 | .9407 | 4.2232 |
|  |  | LPS + 30% | 2.64700^*^ | .56962 | .000 | 1.1173 | 4.1767 |
|  | 30% | Pre | 1.00087 | .66658 | .579 | -.7892 | 2.7910 |
|  |  | LPS Only | 3.58283^*^ | .84159 | .000 | 1.3228 | 5.8429 |
|  |  | LPS + 30% | 3.64786^*^ | .81193 | .000 | 1.4674 | 5.8283 |
|  | LPS Only | Pre | -2.58196^*^ | .61115 | .000 | -4.2232 | -.9407 |
|  |  | 30% | -3.58283^*^ | .84159 | .000 | -5.8429 | -1.3228 |
|  |  | LPS + 30% | .06503 | .76708 | 1.000 | -1.9949 | 2.1250 |
|  | LPS + 30% | Pre | -2.64700^*^ | .56962 | .000 | -4.1767 | -1.1173 |
|  |  | 30% | -3.64786^*^ | .81193 | .000 | -5.8283 | -1.4674 |
|  |  | LPS Only | -.06503 | .76708 | 1.000 | -2.1250 | 1.9949 |
| Dunnett t (2-sided)^b^ | 30% | Pre | 1.00087 | .66658 | .350 | -.6168 | 2.6185 |
|  | LPS Only | Pre | -2.58196^*^ | .61115 | .000 | -4.0651 | -1.0989 |
|  | LPS + 30% | Pre | -2.64700^*^ | .56962 | .000 | -4.0293 | -1.2647 |
| *. The mean difference is significant at the 0.05 level. | | | | | | | |
| b. Dunnett t-tests treat one group as a control, and compare all other groups against it. | | | | | | | |

Analysis of neutrophils in sham animals or animals receiving 15-45% erythrocyte transfusion revealed that levels of these cells did not significantly change compared to the pre-bleed controls when analyzed 5 minutes after transfusion (P =0.53) **(Fig 3B)**

| **Descriptives** | | | | | | | | |
| --- | --- | --- | --- | --- | --- | --- | --- | --- |
| Neutrophils | | | | | | | | |
|  | N | Mean | Std. Deviation | Std. Error | 95% Confidence Interval for Mean | | Minimum | Maximum |
|  |  |  |  |  | Lower Bound | Upper Bound |  |  |
| Pre | 64 | 1140.8125 | 815.85660 | 101.98208 | 937.0176 | 1344.6074 | 246.00 | 4080.00 |
| Sham | 5 | 1571.4000 | 861.10412 | 385.09747 | 502.1980 | 2640.6020 | 561.00 | 2680.00 |
| 15% | 3 | 755.3333 | 226.01180 | 130.48797 | 193.8889 | 1316.7778 | 518.00 | 968.00 |
| 30% | 3 | 1415.3333 | 334.85718 | 193.32989 | 583.5020 | 2247.1647 | 1062.00 | 1728.00 |
| 45% | 3 | 774.6667 | 200.14328 | 115.55278 | 277.4832 | 1271.8501 | 588.00 | 986.00 |
| Total | 78 | 1150.0641 | 783.77318 | 88.74484 | 973.3506 | 1326.7776 | 246.00 | 4080.00 |

| **Test of Homogeneity of Variances** | | | | | |
| --- | --- | --- | --- | --- | --- |
|  | | Levene Statistic | df1 | df2 | Sig. |
| Neutrophils | Based on Mean | 1.336 | 4 | 73 | .265 |
|  | Based on Median | .815 | 4 | 73 | .520 |
|  | Based on Median and with adjusted df | .815 | 4 | 66.955 | .520 |
|  | Based on trimmed mean | 1.084 | 4 | 73 | .371 |

| **ANOVA** | | | | | |
| --- | --- | --- | --- | --- | --- |
| Neutrophils | | | | | |
|  | Sum of Squares | df | Mean Square | F | Sig. |
| Between Groups | 1994407.729 | 4 | 498601.932 | .803 | .527 |
| Within Groups | 45306722.950 | 73 | 620640.040 |  |  |
| Total | 47301130.679 | 77 |  |  |  |

| **Robust Tests of Equality of Means** | | | | |
| --- | --- | --- | --- | --- |
| Neutrophils | | | | |
|  | Statistic^a^ | df1 | df2 | Sig. |
| Welch | 3.076 | 4 | 6.639 | .097 |
| a. Asymptotically F distributed. | | | | |

| **Multiple Comparisons** | | | | | | |
| --- | --- | --- | --- | --- | --- | --- |
| Dependent Variable: Neutrophils | | | | | | |
| Dunnett t (2-sided)^a^ | | | | | | |
| (I) Class1 | (J) Class1 | Mean Difference (I-J) | Std. Error | Sig. | 95% Confidence Interval | |
|  |  |  |  |  | Lower Bound | Upper Bound |
| Sham | Pre | 430.58750 | 365.82169 | .665 | -502.4836 | 1363.6586 |
| 15% | Pre | -385.47917 | 465.37889 | .875 | -1572.4826 | 801.5243 |
| 30% | Pre | 274.52083 | 465.37889 | .960 | -912.4826 | 1461.5243 |
| 45% | Pre | -366.14583 | 465.37889 | .894 | -1553.1493 | 820.8576 |
| a. Dunnett t-tests treat one group as a control, and compare all other groups against it. | | | | | | |

In contrast, at 4 hours post-transfusion, animals receiving 15-45% erythrocyte transfusion had significantly increased number of neutrophils in circulation compared to time 0 controls (P<0.001). **(Fig 3B)**

| **Descriptives** | | | | | | | | |
| --- | --- | --- | --- | --- | --- | --- | --- | --- |
| Neutrophils | | | | | | | | |
|  | N | Mean | Std. Deviation | Std. Error | 95% Confidence Interval for Mean | | Minimum | Maximum |
|  |  |  |  |  | Lower Bound | Upper Bound |  |  |
| Pre | 64 | 1140.8125 | 815.85660 | 101.98208 | 937.0176 | 1344.6074 | 246.00 | 4080.00 |
| Sham | 8 | 1946.5000 | 667.16201 | 235.87739 | 1388.7386 | 2504.2614 | 837.00 | 3080.00 |
| 15% | 6 | 3363.8333 | 1475.50648 | 602.37300 | 1815.3842 | 4912.2824 | 1536.00 | 5610.00 |
| 30% | 9 | 3539.5556 | 1837.99919 | 612.66640 | 2126.7443 | 4952.3668 | 648.00 | 7227.00 |
| 45% | 6 | 4619.5000 | 1903.05762 | 776.92002 | 2622.3635 | 6616.6365 | 2530.00 | 7425.00 |
| Total | 93 | 1810.1075 | 1541.62181 | 159.85874 | 1492.6143 | 2127.6008 | 246.00 | 7425.00 |

| **Test of Homogeneity of Variances** | | | | | |
| --- | --- | --- | --- | --- | --- |
|  | | Levene Statistic | df1 | df2 | Sig. |
| Neutrophils | Based on Mean | 5.658 | 4 | 88 | .000 |
|  | Based on Median | 3.077 | 4 | 88 | .020 |
|  | Based on Median and with adjusted df | 3.077 | 4 | 53.116 | .024 |
|  | Based on trimmed mean | 5.334 | 4 | 88 | .001 |

| **ANOVA** | | | | | |
| --- | --- | --- | --- | --- | --- |
| Neutrophils | | | | | |
|  | Sum of Squares | df | Mean Square | F | Sig. |
| Between Groups | 117577410.619 | 4 | 29394352.655 | 25.593 | .000 |
| Within Groups | 101069588.306 | 88 | 1148518.049 |  |  |
| Total | 218646998.925 | 92 |  |  |  |

| **Robust Tests of Equality of Means** | | | | |
| --- | --- | --- | --- | --- |
| Neutrophils | | | | |
|  | Statistic^a^ | df1 | df2 | Sig. |
| Welch | 11.240 | 4 | 13.127 | .000 |
| a. Asymptotically F distributed. | | | | |

| **Multiple Comparisons** | | | | | | |
| --- | --- | --- | --- | --- | --- | --- |
| Dependent Variable: Neutrophils | | | | | | |
| Dunnett t (2-sided)^a^ | | | | | | |
| (I) Class1 | (J) Class1 | Mean Difference (I-J) | Std. Error | Sig. | 95% Confidence Interval | |
|  |  |  |  |  | Lower Bound | Upper Bound |
| Sham | Pre | 805.68750 | 401.88350 | .176 | -214.5991 | 1825.9741 |
| 15% | Pre | 2223.02083^*^ | 457.56450 | .000 | 1061.3735 | 3384.6682 |
| 30% | Pre | 2398.74306^*^ | 381.52157 | .000 | 1430.1506 | 3367.3355 |
| 45% | Pre | 3478.68750^*^ | 457.56450 | .000 | 2317.0401 | 4640.3349 |
| *. The mean difference is significant at the 0.05 level. | | | | | | |
| a. Dunnett t-tests treat one group as a control, and compare all other groups against it. | | | | | | |

When the groups receiving LPS alone or LPS+30% mismatched erythrocytes were compared to animals receiving the 30% transfusion alone, a significant reduction of neutrophils (P = 0.009 and P < 0.001, respectively), was observed at 4 hours **(Fig 3B)**

| **Descriptives** | | | | | | | | |
| --- | --- | --- | --- | --- | --- | --- | --- | --- |
| Neutrophils | | | | | | | | |
|  | N | Mean | Std. Deviation | Std. Error | 95% Confidence Interval for Mean | | Minimum | Maximum |
|  |  |  |  |  | Lower Bound | Upper Bound |  |  |
| Pre | 64 | 1140.8125 | 815.85660 | 101.98208 | 937.0176 | 1344.6074 | 246.00 | 4080.00 |
| 30% | 9 | 3539.5556 | 1837.99919 | 612.66640 | 2126.7443 | 4952.3668 | 648.00 | 7227.00 |
| LPS Only | 11 | 2056.9091 | 1135.53824 | 342.37766 | 1294.0441 | 2819.7741 | 986.00 | 4071.00 |
| LPS + 30% | 13 | 1394.9231 | 997.99077 | 276.79284 | 791.8433 | 1998.0029 | 16.00 | 3657.00 |
| Total | 97 | 1501.3196 | 1220.72591 | 123.94594 | 1255.2888 | 1747.3503 | 16.00 | 7227.00 |

| **Test of Homogeneity of Variances** | | | | | |
| --- | --- | --- | --- | --- | --- |
|  | | Levene Statistic | df1 | df2 | Sig. |
| Neutrophils | Based on Mean | 4.152 | 3 | 93 | .008 |
|  | Based on Median | 2.093 | 3 | 93 | .106 |
|  | Based on Median and with adjusted df | 2.093 | 3 | 61.572 | .110 |
|  | Based on trimmed mean | 4.003 | 3 | 93 | .010 |

| **ANOVA** | | | | | |
| --- | --- | --- | --- | --- | --- |
| Neutrophils | | | | | |
|  | Sum of Squares | df | Mean Square | F | Sig. |
| Between Groups | 49250075.288 | 3 | 16416691.763 | 16.276 | .000 |
| Within Groups | 93806411.804 | 93 | 1008671.095 |  |  |
| Total | 143056487.093 | 96 |  |  |  |

| **Robust Tests of Equality of Means** | | | | |
| --- | --- | --- | --- | --- |
| Neutrophils | | | | |
|  | Statistic^a^ | df1 | df2 | Sig. |
| Welch | 6.457 | 3 | 18.332 | .004 |
| a. Asymptotically F distributed. | | | | |

| **Multiple Comparisons** | | | | | | | |
| --- | --- | --- | --- | --- | --- | --- | --- |
| Dependent Variable: Neutrophils | | | | | | | |
|  | (I) Class30 | (J) Class30 | Mean Difference (I-J) | Std. Error | Sig. | 95% Confidence Interval | |
|  |  |  |  |  |  | Lower Bound | Upper Bound |
| Bonferroni | Pre | 30% | -2398.74306^*^ | 357.54028 | .000 | -3362.6230 | -1434.8631 |
|  |  | LPS Only | -916.09659^*^ | 327.80765 | .038 | -1799.8214 | -32.3718 |
|  |  | LPS + 30% | -254.11058 | 305.53326 | 1.000 | -1077.7866 | 569.5655 |
|  | 30% | Pre | 2398.74306^*^ | 357.54028 | .000 | 1434.8631 | 3362.6230 |
|  |  | LPS Only | 1482.64646^*^ | 451.41105 | .009 | 265.7037 | 2699.5892 |
|  |  | LPS + 30% | 2144.63248^*^ | 435.50505 | .000 | 970.5702 | 3318.6948 |
|  | LPS Only | Pre | 916.09659^*^ | 327.80765 | .038 | 32.3718 | 1799.8214 |
|  |  | 30% | -1482.64646^*^ | 451.41105 | .009 | -2699.5892 | -265.7037 |
|  |  | LPS + 30% | 661.98601 | 411.44557 | .666 | -447.2152 | 1771.1872 |
|  | LPS + 30% | Pre | 254.11058 | 305.53326 | 1.000 | -569.5655 | 1077.7866 |
|  |  | 30% | -2144.63248^*^ | 435.50505 | .000 | -3318.6948 | -970.5702 |
|  |  | LPS Only | -661.98601 | 411.44557 | .666 | -1771.1872 | 447.2152 |
| Hochberg | Pre | 30% | -2398.74306^*^ | 357.54028 | .000 | -3358.9127 | -1438.5735 |
|  |  | LPS Only | -916.09659^*^ | 327.80765 | .037 | -1796.4196 | -35.7736 |
|  |  | LPS + 30% | -254.11058 | 305.53326 | .955 | -1074.6160 | 566.3949 |
|  | 30% | Pre | 2398.74306^*^ | 357.54028 | .000 | 1438.5735 | 3358.9127 |
|  |  | LPS Only | 1482.64646^*^ | 451.41105 | .009 | 270.3882 | 2694.9048 |
|  |  | LPS + 30% | 2144.63248^*^ | 435.50505 | .000 | 975.0895 | 3314.1754 |
|  | LPS Only | Pre | 916.09659^*^ | 327.80765 | .037 | 35.7736 | 1796.4196 |
|  |  | 30% | -1482.64646^*^ | 451.41105 | .009 | -2694.9048 | -270.3882 |
|  |  | LPS + 30% | 661.98601 | 411.44557 | .500 | -442.9455 | 1766.9175 |
|  | LPS + 30% | Pre | 254.11058 | 305.53326 | .955 | -566.3949 | 1074.6160 |
|  |  | 30% | -2144.63248^*^ | 435.50505 | .000 | -3314.1754 | -975.0895 |
|  |  | LPS Only | -661.98601 | 411.44557 | .500 | -1766.9175 | 442.9455 |
| Dunnett t (2-sided)^b^ | 30% | Pre | 2398.74306^*^ | 357.54028 | .000 | 1531.0832 | 3266.4029 |
|  | LPS Only | Pre | 916.09659^*^ | 327.80765 | .019 | 120.5903 | 1711.6029 |
|  | LPS + 30% | Pre | 254.11058 | 305.53326 | .786 | -487.3414 | 995.5626 |
| *. The mean difference is significant at the 0.05 level. | | | | | | | |
| b. Dunnett t-tests treat one group as a control, and compare all other groups against it. | | | | | | | |

In contrast, animals receiving LPS+30% transfusion demonstrated a significant increase in lung damage compared to the other groups including animals treated with LPS alone (P < 0.05) **(Fig 5)**

| **Hypothesis Test Summary** | | | | |
| --- | --- | --- | --- | --- |
|  | Null Hypothesis | Test | Sig. | Decision |
| 1 | The distribution of damage is the same across categories of Group. | Independent-Samples Kruskal-Wallis Test | .000 | Retain the null hypothesis. |
| Asymptotic significances are displayed. The significance level is .050. | | | | |

| **Independent-Samples Kruskal-Wallis Test Summary** | |
| --- | --- |
| Total N | 115 |
| Test Statistic | 23.150^a^ |
| Degree Of Freedom | 5 |
| Asymptotic Sig.(2-sided test) | .000 |
| a. The test statistic is adjusted for ties. | |

| **Pairwise Comparisons of Group** | | | | | |
| --- | --- | --- | --- | --- | --- |
| Sample 1-Sample 2 | Test Statistic | Std. Error | Std. Test Statistic | Sig. | Adj. Sig.^a^ |
| 30%-sham | 2.025 | 8.895 | .228 | .820 | 1.000 |
| 30%-lps | 5.500 | 9.744 | .564 | .572 | 1.000 |
| 30%-45% | -12.200 | 9.744 | -1.252 | .211 | 1.000 |
| 30%-15% | 12.833 | 9.744 | 1.317 | .188 | 1.000 |
| 30%-lps30% | 39.475 | 8.895 | 4.438 | .000 | .000 |
| sham -lps | -3.475 | 10.525 | -.330 | .741 | 1.000 |
| sham -45% | -10.175 | 10.525 | -.967 | .334 | 1.000 |
| sham -15% | -10.808 | 10.525 | -1.027 | .304 | 1.000 |
| sham -lps30% | -37.450 | 9.744 | -3.843 | .000 | .002 |
| lps-45% | -6.700 | 11.251 | -.595 | .552 | 1.000 |
| lps-15% | -7.333 | 11.251 | -.652 | .515 | 1.000 |
| lps-lps30% | -33.975 | 10.525 | -3.228 | .001 | .019 |
| 45%-15% | .633 | 11.251 | .056 | .955 | 1.000 |
| 45%-lps30% | 27.275 | 10.525 | 2.592 | .010 | .033 |
| 15%-lps30% | 26.642 | 10.525 | 2.531 | .011 | .042 |
| Each row tests the null hypothesis that the Sample 1 and Sample 2 distributions are the same.  Asymptotic significances (2-sided tests) are displayed. The significance level is .05. | | | | | |
| a. Significance values have been adjusted by the Bonferroni correction for multiple tests. | | | | | |

Wet lung weight showed that animals receiving 30% erythrocyte transfusion, LPS alone or LPS+30% transfusion had a significant increase in lung weight of 34%, 36% and 41% compared with sham (P = 0.008, P = 0.009, P = 0.001, respectively) **(Fig 6A)**

| **Hypothesis Test Summary** | | | | |
| --- | --- | --- | --- | --- |
|  | Null Hypothesis | Test | Sig. | Decision |
| 1 | The distribution of Lung Weight (g) is the same across categories of Group. | Independent-Samples Kruskal-Wallis Test | .003 | Reject the null hypothesis. |
| Asymptotic significances are displayed. The significance level is .050. | | | | |

| **Independent-Samples Kruskal-Wallis Test Summary** | |
| --- | --- |
| Total N | 36 |
| Test Statistic | 17.675^a^ |
| Degree Of Freedom | 5 |
| Asymptotic Sig.(2-sided test) | .003 |
| a. The test statistic is adjusted for ties. | |

| **Pairwise Comparisons of Group** | | | | | |
| --- | --- | --- | --- | --- | --- |
| Sample 1-Sample 2 | Test Statistic | Std. Error | Std. Test Statistic | Sig. | Adj. Sig.^a^ |
| 45%-sham | .417 | 7.447 | .056 | .955 | 1.000 |
| 45%-15% | 5.667 | 8.599 | .659 | .510 | 1.000 |
| 45%-lps | 15.762 | 7.267 | 2.169 | .030 | .451 |
| 45%-30% | 16.667 | 7.447 | 2.238 | .025 | .378 |
| 45%-lps30% | 17.833 | 6.860 | 2.600 | .009 | .140 |
| sham -15% | -5.250 | 7.447 | -.705 | .481 | 1.000 |
| sham - sham | -15.345 | 5.859 | -2.619 | .009 | .032 |
| sham -30% | -16.250 | 6.080 | -2.673 | .008 | .022 |
| sham - lps30% | -17.417 | 5.345 | -3.259 | .001 | .017 |
| 15%- sham | 10.095 | 7.267 | 1.389 | .165 | 1.000 |
| 15%-30% | -11.000 | 7.447 | -1.477 | .140 | 1.000 |
| 15%- lps30% | 12.167 | 6.860 | 1.774 | .076 | 1.000 |
| sham -30% | -.905 | 5.859 | -.154 | .877 | 1.000 |
| sham - lps30% | -2.071 | 5.092 | -.407 | .684 | 1.000 |
| 30%- lps30% | 1.167 | 5.345 | .218 | .827 | 1.000 |
| Each row tests the null hypothesis that the Sample 1 and Sample 2 distributions are the same.  Asymptotic significances (2-sided tests) are displayed. The significance level is .05. | | | | | |
| a. Significance values have been adjusted by the Bonferroni correction for multiple tests. | | | | | |

LPS treated animals showed no increase in dry:wet lung weight compared to sham, whereas animals receiving LPS+30% erythrocyte transfusion showed a significant increase in dry:wet ratio over animals treated with LPS only (P = 0.035) **(Fig 6B)**

| **Multiple Comparisons** | | | | | | |
| --- | --- | --- | --- | --- | --- | --- |
| Dependent Variable: dry:wet | | | | | | |
| Dunnett t (2-sided)^a^ | | | | | | |
| (I) Group | (J) Group | Mean Difference (I-J) | Std. Error | Sig. | 95% Confidence Interval | |
|  |  |  |  |  | Lower Bound | Upper Bound |
| lps | sham | -.00083 | .00994 | .995 | -.0263 | .0246 |
| lps30% | sham | .02250 | .00994 | .084 | -.0030 | .0480 |
| a. Dunnett t-tests treat one group as a control, and compare all other groups against it. | | | | | | |

| **Multiple Comparisons** | | | | | | |
| --- | --- | --- | --- | --- | --- | --- |
| Dependent Variable: dry:wet | | | | | | |
| Dunnett t (2-sided)^a^ | | | | | | |
| (I) Group | (J) Group | Mean Difference (I-J) | Std. Error | Sig. | 95% Confidence Interval | |
|  |  |  |  |  | Lower Bound | Upper Bound |
| sham | lps30% | -.02250 | .00994 | .076 | -.0475 | .0025 |
| lps | lps30% | -.02333 | .00448 | .035 | -.047263 | -.00404 |
| a. Dunnett t-tests treat one group as a control, and compare all other groups against it. | | | | | | |

With the exception of time 0, rats treated with LPS alone showed a significant increase in C5a levels over sham animals at all other time points (5-240 minutes) (P ≤ 0.001). Animals receiving LPS+30% erythrocyte transfusion showed significantly enhanced C5a accumulation over sham animals (P < 0.001) and animals treated with LPS only at all time points (5-240 minutes) (P ≤ 0.042) **(Fig 7)**

| **Parameter Estimates** | | | | | | | |
| --- | --- | --- | --- | --- | --- | --- | --- |
| Parameter | B | Std. Error | 95% Wald Confidence Interval | | Hypothesis Test | | |
|  |  |  | Lower | Upper | Wald Chi-Square | df | Sig. |
| (Intercept) | 2014.167 | 73.0119 | 1871.066 | 2157.267 | 761.034 | 1 | .000 |
| [Time=.00] | -1887.333 | 103.2544 | -2089.708 | -1684.959 | 334.103 | 1 | .000 |
| [Time=.08] | -1736.333 | 103.2544 | -1938.708 | -1533.959 | 282.780 | 1 | .000 |
| [Time=1.00] | -380.833 | 103.2544 | -583.208 | -178.459 | 13.604 | 1 | .000 |
| [Time=2.00] | 265.333 | 103.2544 | 62.959 | 467.708 | 6.603 | 1 | .010 |
| [Time=3.00] | 469.167 | 103.2544 | 266.792 | 671.541 | 20.646 | 1 | .000 |
| [Time=4.00] | 0^a^ | . | . | . | . | . | . |
| [name=sham]([Time=.00]) | -34.403 | 103.2544 | -236.778 | 167.971 | .111 | 1 | .739 |
| [name=lps]([Time=.00]) | 0^a^ | . | . | . | . | . | . |
| [name= sham]([Time=.08]) | -179.745 | 103.2544 | -382.120 | 22.630 | 3.030 | 1 | .082 |
| [name= lps]([Time=.08]) | 0^a^ | . | . | . | . | . | . |
| [name= sham]([Time=1.00]) | -1529.492 | 103.2544 | -1731.866 | -1327.117 | 219.421 | 1 | .000 |
| [name= lps]([Time=1.00]) | 0^a^ | . | . | . | . | . | . |
| [name= sham]([Time=2.00]) | -2227.182 | 103.2544 | -2429.556 | -2024.807 | 465.259 | 1 | .000 |
| [name= lps]([Time=2.00]) | 0^a^ | . | . | . | . | . | . |
| [name= sham]([Time=3.00]) | -2441.665 | 103.2544 | -2644.040 | -2239.290 | 559.185 | 1 | .000 |
| [name= lps]([Time=3.00]) | 0^a^ | . | . | . | . | . | . |
| [name= sham]([Time=4.00]) | -1976.085 | 103.2544 | -2178.460 | -1773.710 | 366.264 | 1 | .000 |
| [name= lps]([Time=4.00]) | 0^a^ | . | . | . | . | . | . |
| (Scale) | 31984.389^b^ | 5330.7316 | 23071.251 | 44340.950 |  |  |  |
| Dependent Variable: c5a  Model: (Intercept), Time, name(Time) | | | | | | | |
| a. Set to zero because this parameter is redundant. | | | | | | | |
| b. Maximum likelihood estimate. | | | | | | | |

| **Parameter Estimates** | | | | | | | |
| --- | --- | --- | --- | --- | --- | --- | --- |
| Parameter | B | Std. Error | 95% Wald Confidence Interval | | Hypothesis Test | | |
|  |  |  | Lower | Upper | Wald Chi-Square | df | Sig. |
| (Intercept) | 5679.250 | 414.3541 | 4867.131 | 6491.369 | 187.862 | 1 | .000 |
| [Time=.00] | -5635.750 | 585.9851 | -6784.260 | -4487.240 | 92.498 | 1 | .000 |
| [Time=.08] | -4981.050 | 555.9143 | -6070.622 | -3891.478 | 80.283 | 1 | .000 |
| [Time=1.00] | -2471.250 | 555.9143 | -3560.822 | -1381.678 | 19.761 | 1 | .000 |
| [Time=2.00] | -657.250 | 585.9851 | -1805.760 | 491.260 | 1.258 | 1 | .262 |
| [Time=3.00] | 362.000 | 585.9851 | -786.510 | 1510.510 | .382 | 1 | .537 |
| [Time=4.00] | 0^a^ | . | . | . | . | . | . |
| [name=sham]([Time=.00]) | 48.930 | 534.9288 | -999.511 | 1097.371 | .008 | 1 | .927 |
| [name=lps30%]([Time=.00]) | 0^a^ | . | . | . | . | . | . |
| [name= sham]([Time=.08]) | -600.112 | 501.8077 | -1583.637 | 383.413 | 1.430 | 1 | .232 |
| [name= lps30%]([Time=.08]) | 0^a^ | . | . | . | . | . | . |
| [name= sham]([Time=1.00]) | -3104.158 | 501.8077 | -4087.683 | -2120.633 | 38.266 | 1 | .000 |
| [name= lps30%]([Time=1.00]) | 0^a^ | . | . | . | . | . | . |
| [name= sham]([Time=2.00]) | -4969.682 | 534.9288 | -6018.123 | -3921.240 | 86.311 | 1 | .000 |
| [name= lps30%]([Time=2.00]) | 0^a^ | . | . | . | . | . | . |
| [name= sham]([Time=3.00]) | -5999.582 | 534.9288 | -7048.023 | -4951.140 | 125.791 | 1 | .000 |
| [name= lps30%]([Time=3.00]) | 0^a^ | . | . | . | . | . | . |
| [name= sham ([Time=4.00]) | -5641.168 | 534.9288 | -6689.610 | -4592.727 | 111.211 | 1 | .000 |
| [name= lps30%]([Time=4.00]) | 0^a^ | . | . | . | . | . | . |
| (Scale) | 686757.172^b^ | 123345.2293 | 482973.045 | 976525.332 |  |  |  |
| Dependent Variable: c5a  Model: (Intercept), Time, name(Time) | | | | | | | |
| a. Set to zero because this parameter is redundant. | | | | | | | |
| b. Maximum likelihood estimate. | | | | | | | |

| **Parameter Estimates** | | | | | | | |
| --- | --- | --- | --- | --- | --- | --- | --- |
| Parameter | B | Std. Error | 95% Wald Confidence Interval | | Hypothesis Test | | |
|  |  |  | Lower | Upper | Wald Chi-Square | df | Sig. |
| (Intercept) | 5679.250 | 425.2653 | 4845.745 | 6512.755 | 178.345 | 1 | .000 |
| [Time=.00] | -5635.750 | 601.4159 | -6814.504 | -4456.996 | 87.812 | 1 | .000 |
| [Time=.08] | -4981.050 | 570.5533 | -6099.314 | -3862.786 | 76.217 | 1 | .000 |
| [Time=1.00] | -2471.250 | 570.5533 | -3589.514 | -1352.986 | 18.760 | 1 | .000 |
| [Time=2.00] | -657.250 | 601.4159 | -1836.004 | 521.504 | 1.194 | 1 | .274 |
| [Time=3.00] | 362.000 | 601.4159 | -816.754 | 1540.754 | .362 | 1 | .547 |
| [Time=4.00] | 0^a^ | . | . | . | . | . | . |
| [name=lps]([Time=.00]) | 83.333 | 549.0151 | -992.717 | 1159.383 | .023 | 1 | .879 |
| [name=lps30%]([Time=.00]) | 0^a^ | . | . | . | . | . | . |
| [name= lps]([Time=.08]) | -420.367 | 515.0219 | -1429.791 | 589.058 | .666 | 1 | .414 |
| [name= lps30%]([Time=.08]) | 0^a^ | . | . | . | . | . | . |
| [name= lps]([Time=1.00]) | -1574.667 | 515.0219 | -2584.091 | -565.242 | 9.348 | 1 | .042 |
| [name= lps30%]([Time=1.00]) | 0^a^ | . | . | . | . | . | . |
| [name= lps]([Time=2.00]) | -2742.500 | 549.0151 | -3818.550 | -1666.450 | 24.953 | 1 | .000 |
| [name= lps30%]([Time=2.00]) | 0^a^ | . | . | . | . | . | . |
| [name= lps]([Time=3.00]) | -3557.917 | 549.0151 | -4633.967 | -2481.867 | 41.997 | 1 | .000 |
| [name= lps30%]([Time=3.00]) | 0^a^ | . | . | . | . | . | . |
| [name= lps]([Time=4.00]) | -3665.083 | 549.0151 | -4741.133 | -2589.033 | 44.566 | 1 | .000 |
| [name= lps30%]([Time=4.00]) | 0^a^ | . | . | . | . | . | . |
| (Scale) | 723402.290^b^ | 129926.8867 | 508744.315 | 1028632.376 |  |  |  |
| Dependent Variable: c5a  Model: (Intercept), Time, name(Time) | | | | | | | |
| a. Set to zero because this parameter is redundant. | | | | | | | |
| b. Maximum likelihood estimate. | | | | | | | |

Compared to sham, LPS only, or animals receiving 15, 30 and 45% erythrocyte transfusion, rats receiving LPS+30% transfusion had significant increases in free DNA levels at 60-240 minutes compared to all other groups (P ≤ 0.001) **(Fig 8)**

| **Parameter Estimates** | | | | | | | |
| --- | --- | --- | --- | --- | --- | --- | --- |
| Parameter | B | Std. Error | 95% Wald Confidence Interval | | Hypothesis Test | | |
|  |  |  | Lower | Upper | Wald Chi-Square | df | Sig. |
| (Intercept) | 11491.333 | 646.8495 | 10223.532 | 12759.135 | 315.598 | 1 | .000 |
| [Time=.00] | -11452.333 | 959.4329 | -13332.787 | -9571.879 | 142.482 | 1 | .000 |
| [Time=.08] | -11256.000 | 914.7833 | -13048.942 | -9463.058 | 151.402 | 1 | .000 |
| [Time=1.00] | -8018.833 | 914.7833 | -9811.776 | -6225.891 | 76.840 | 1 | .000 |
| [Time=2.00] | -2169.167 | 914.7833 | -3962.109 | -376.224 | 5.623 | 1 | .018 |
| [Time=3.00] | -1239.333 | 914.7833 | -3032.276 | 553.609 | 1.835 | 1 | .175 |
| [Time=4.00] | 0^a^ | . | . | . | . | . | . |
| [Group=sham]([Time=.00]) | 32.333 | 959.4329 | -1848.121 | 1912.787 | .001 | 1 | .973 |
| [Group=15%]([Time=.00]) | 66.000 | 959.4329 | -1814.454 | 1946.454 | .005 | 1 | .945 |
| [Group=30%]([Time=.00]) | -7.333 | 959.4329 | -1887.787 | 1873.121 | .000 | 1 | .994 |
| [Group=45%]([Time=.00]) | 49.500 | 959.4329 | -1830.954 | 1929.954 | .003 | 1 | .959 |
| [Group=lps]([Time=.00]) | 56.200 | 1002.0949 | -1907.870 | 2020.270 | .003 | 1 | .955 |
| [Group= lps30%]([Time=.00]) | 0^a^ | . | . | . | . | . | . |
| [Group= sham]]([Time=.08]) | -201.333 | 914.7833 | -1994.276 | 1591.609 | .048 | 1 | .826 |
| [Group=15%]([Time=.08]) | -173.933 | 959.4329 | -2054.387 | 1706.521 | .033 | 1 | .856 |
| [Group=30%]([Time=.08]) | -189.133 | 959.4329 | -2069.587 | 1691.321 | .039 | 1 | .844 |
| [Group=45%]([Time=.08]) | -36.733 | 959.4329 | -1917.187 | 1843.721 | .001 | 1 | .969 |
| [Group= lps]([Time=.08]) | -110.500 | 914.7833 | -1903.442 | 1682.442 | .015 | 1 | .904 |
| [Group= lps30%]([Time=.08]) | 0^a^ | . | . | . | . | . | . |
| [Group= sham]]([Time=1.00]) | -3438.500 | 914.7833 | -5231.442 | -1645.558 | 14.129 | 1 | .000 |
| [Group=15%]([Time=1.00]) | -3441.700 | 959.4329 | -5322.154 | -1561.246 | 12.868 | 1 | .000 |
| [Group=30%]([Time=1.00]) | -3447.500 | 959.4329 | -5327.954 | -1567.046 | 12.912 | 1 | .000 |
| [Group=45%]([Time=1.00]) | -3093.000 | 914.7833 | -4885.942 | -1300.058 | 11.432 | 1 | .001 |
| [Group= lps]([Time=1.00]) | -3080.333 | 914.7833 | -4873.276 | -1287.391 | 11.339 | 1 | .001 |
| [Group= lps30%]([Time=1.00]) | 0^a^ | . | . | . | . | . | . |
| [Group= sham]]([Time=2.00]) | -9295.833 | 914.7833 | -11088.776 | -7502.891 | 103.262 | 1 | .000 |
| [Group=15%]([Time=2.00]) | -9287.167 | 914.7833 | -11080.109 | -7494.224 | 103.069 | 1 | .000 |
| [Group=30%]([Time=2.00]) | -9282.000 | 914.7833 | -11074.942 | -7489.058 | 102.955 | 1 | .000 |
| [Group=45%]([Time=2.00]) | -9110.917 | 1022.7589 | -11115.487 | -7106.346 | 79.356 | 1 | .000 |
| [Group= lps]([Time=2.00]) | -8810.333 | 914.7833 | -10603.276 | -7017.391 | 92.757 | 1 | .000 |
| [Group= lps30%]([Time=2.00]) | 0^a^ | . | . | . | . | . | . |
| [Group= sham]]([Time=3.00]) | -10221.833 | 914.7833 | -12014.776 | -8428.891 | 124.859 | 1 | .000 |
| [Group=15%]([Time=3.00]) | -10205.250 | 1022.7589 | -12209.821 | -8200.679 | 99.564 | 1 | .000 |
| [Group=30%]([Time=3.00]) | -10207.667 | 914.7833 | -12000.609 | -8414.724 | 124.514 | 1 | .000 |
| [Group=45%]([Time=3.00]) | -9910.800 | 959.4329 | -11791.254 | -8030.346 | 106.706 | 1 | .000 |
| [Group= lps]([Time=3.00]) | -9982.500 | 914.7833 | -11775.442 | -8189.558 | 119.081 | 1 | .000 |
| [Group= lps30%]([Time=3.00]) | 0^a^ | . | . | . | . | . | . |
| [Group= sham]]([Time=4.00]) | -11468.833 | 914.7833 | -13261.776 | -9675.891 | 157.182 | 1 | .000 |
| [Group=15%]([Time=4.00]) | -11447.167 | 914.7833 | -13240.109 | -9654.224 | 156.588 | 1 | .000 |
| [Group=30%]([Time=4.00]) | -11414.833 | 914.7833 | -13207.776 | -9621.891 | 155.705 | 1 | .000 |
| [Group=45%]([Time=4.00]) | -11222.133 | 959.4329 | -13102.587 | -9341.679 | 136.811 | 1 | .000 |
| [Group= lps]([Time=4.00]) | -11342.833 | 914.7833 | -13135.776 | -9549.891 | 153.747 | 1 | .000 |
| [Group=lps30%]([Time=4.00]) | 0^a^ | . | . | . | . | . | . |
| (Scale) | 2510485.637^b^ | 249186.6203 | 2066659.337 | 3049626.042 |  |  |  |
| Dependent Variable: DNA  Model: (Intercept), Time, Group(Time) | | | | | | | |
| a. Set to zero because this parameter is redundant. | | | | | | | |
| b. Maximum likelihood estimate. | | | | | | | |
